# Copper-Mediated Trimerization of Diazopeptides Generates Peptide Derivatives of *Cis*- and *Trans*-Aconitic Acid That Adopt Folded Structures

**DOI:** 10.1021/acs.joc.5c02991

**Published:** 2026-06-01

**Authors:** Timothy P. Curran, Lilly L. Pubillones, Alessandro Marrone, Iogann Tolbatov, Sara C. Ingrey

**Affiliations:** † Department of Chemistry, 3757 Trinity College, 300 Summit Street, Hartford, Connecticut 06106, United States; ‡ Dipartimento di Farmacia, Università degli Studi “G. D’Annunzio” Chieti-Pescara, Via dei Vestini 31, Chieti 66100, Italy; § Department of Chemical, Physical, Mathematical and Natural Sciences, 9312 University of Sassari, Sassari 07100, Italy

## Abstract

The reaction of two diazopeptides with CuI in MeCN produces peptide derivatives of [*E*] and [*Z*]-aconitic acid via a trimerization reaction. Best yields of the trimeric products occur when 0.5 equiv of CuI is used. The conformations of the peptide derivatives of [*E*] and [*Z*]-aconitic acid were probed using computational and NMR methods. Both the isomers adopt a conformation having two intramolecular hydrogen bonds, where two of the peptide chains are parallel to each other, while the other is perpendicular.

## Graphical abstract

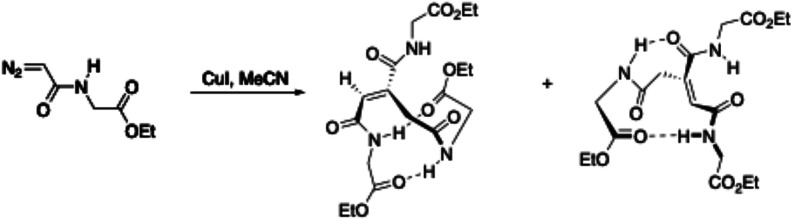

Trimerization reactions are relatively uncommon but notable for how they can be used to quickly construct a complex molecule from simple starting materials. Their greatest utility has been in the synthesis of substituted aromatic rings via [2 + 2 + 2] cycloadditions.^1–7^ In a recent report, Manna and Antonchick reported on a [1 + 1+1] cyclotrimerization to produce cyclopropanes.^8^

In 1968, Saegusa and co-workers reported that the reaction of ethyl diazoacetate (**1a**) with 1-butanol and CuCl yielded several products, one of which was the trimer **2** (Scheme 1).^9^ The reaction could have produced either the *E* or *Z* isomer of **2**, but no structural determination was made. In 1979, Maryanoff reported this same trimerization when **1a** was reacted with *N*-methylpyrrole in the presence of Cu catalysts.^10^ In this work, Maryanoff was able to establish that the reaction produced **2a**, the triethyl ester of (*E*)-aconitic acid (Scheme 1), but not the *Z* isomer. This outcome was also reported in 1990 by Dzhemilev and co-workers, who reacted methyl diazoacetate (**1b**) with Cu­(acac)_2_ to yield **2b**, the trimethyl ester of (*E*)-aconitic acid (Scheme 1); again, the *Z* isomer was not reported.^11^

**Scheme 1:**
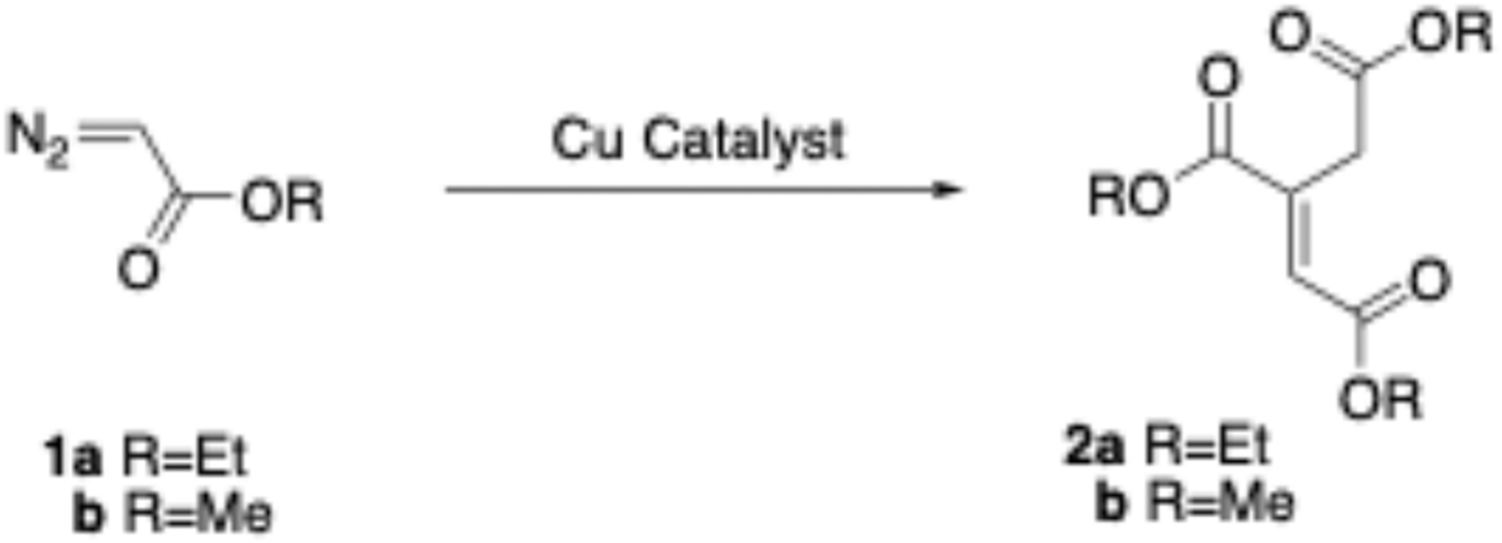
Trimerization Reactions of Diazoesters

In the course of studies aimed at preparing and studying an organometallic β-sheet model,^12^ we sought to couple the ferrocenyldialkyne (**3**)^13^ with two equivalents of diazopeptide (**4**)^14^ to yield **5** using the Cu-catalyzed method developed by Suárez and Fu^15^ (Scheme 2). In our hands, this reaction would not go to completion unless we used a large excess of **4**. Because the crude product did not show the presence of any unreacted **4**, we surmised that the Cu was catalyzing a separate reaction of **4**. We tested this hypothesis and discovered that **4** and a structural relative undergo a trimerization reaction to yield peptide derivatives of *cis*- and *trans*-aconitic acid, which adopt compact, folded structures. Details about these investigations are provided below.

**Scheme 2:**
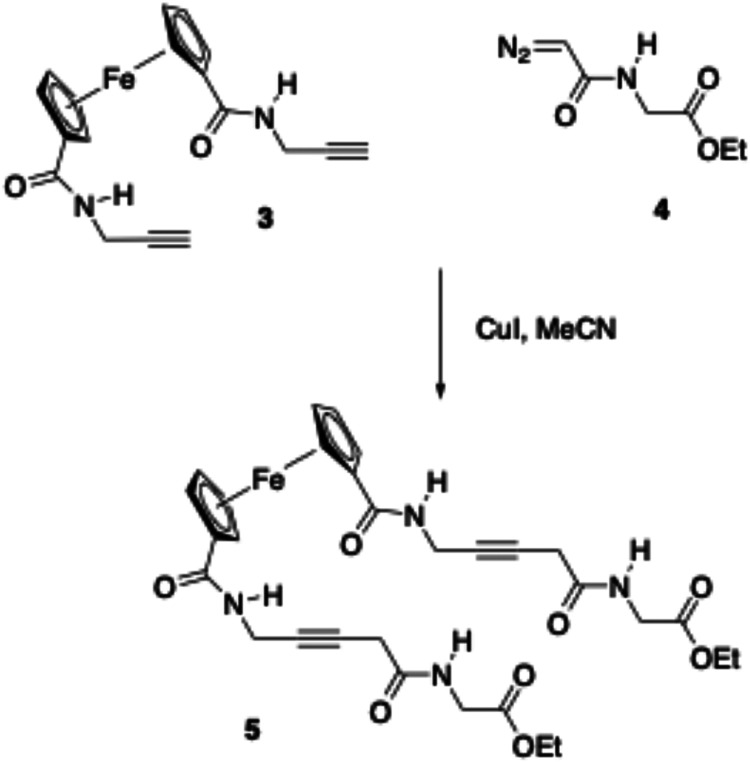
Cu-Mediated Coupling of a Ferrocenyldialkyne with a Diazopeptide

To determine what reaction(s) were in competition with the desired coupling to the alkynes, the diazoglycine peptide, **4** (prepared via reaction of commercially available ethyl ester of glycine–glycine with HONO),^14^ was reacted with CuI in MeCN over 24 h at 23 °C. Two major products were produced; they were isolated pure by flash chromatography and then analyzed by mass spectrometry and NMR spectroscopy (Scheme 3). The first product to elute was identified as the (*E*)-isomer, **6**, while the second product to elute was identified as the (*Z*)-isomer **7**. The structures of **6** and **7** were assigned on the basis of their ^1^H NMR spectra. The alkene CH in (*Z*)-aconitic acid appears at 5.65 ppm,^16^ while the alkene CH in (*E*)-aconitic acid appears at 6.8 ppm.^17^ Similar differences in the alkene CH chemical shift are also seen between fumaric and maleic acids. For trimer **6**, the alkene CH appears at 6.89 ppm, identifying it as a peptide derivative of (*E*)-aconitic acid, while for trimer **7**, the alkene CH appears at 6.21 ppm, identifying it as a peptide derivative of (*Z*)-aconitic acid.

**Scheme 3:**
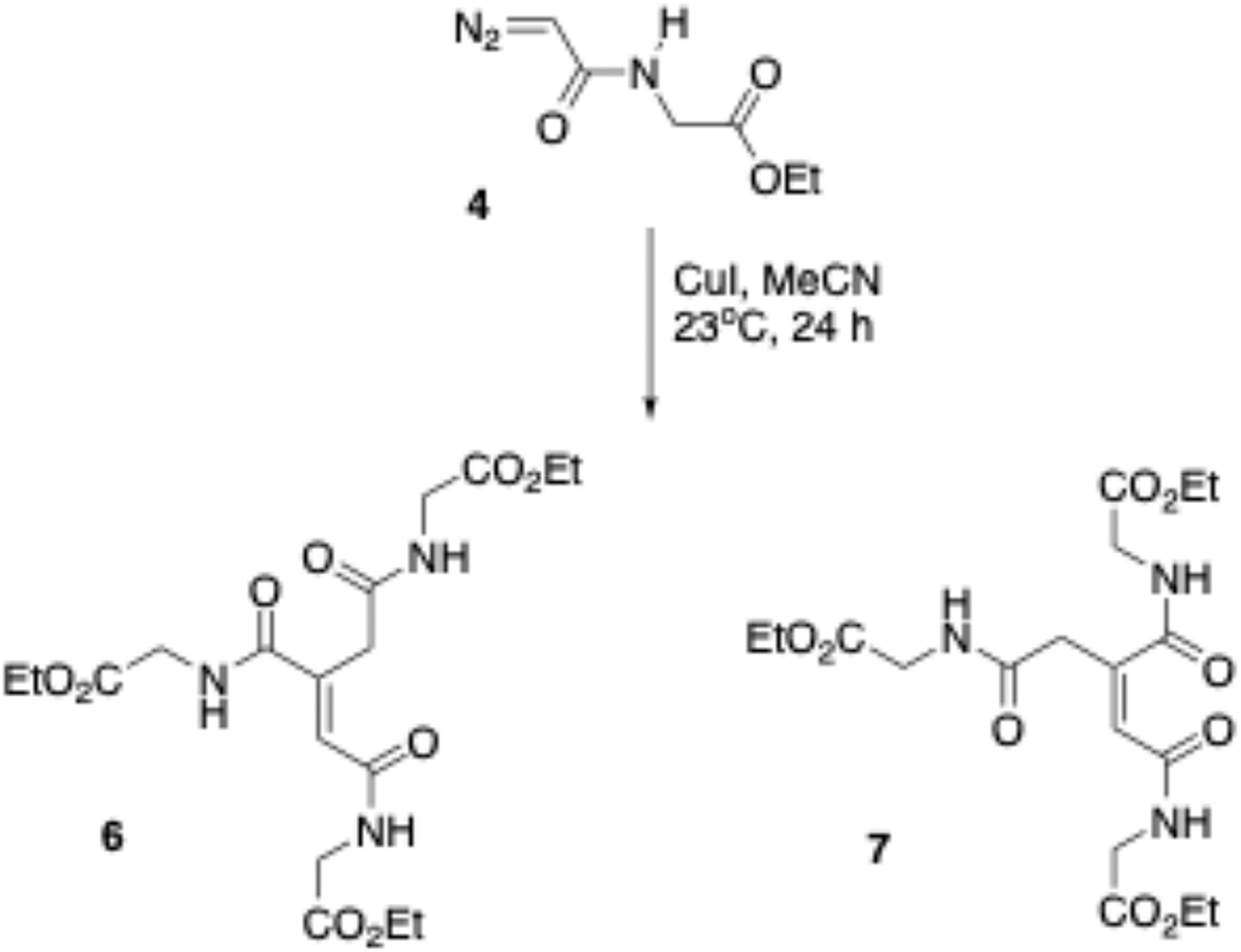
Trimerization Products (**6** and **7**) Obtained from **4**, a Glycine Diazopeptide

To further investigate this reaction, the alanine diazopeptide (**8)** was prepared following a literature procedure^18^ and then reacted with CuI (Scheme 4). When the reaction was done using 10% CuI, four products were isolated and identified as dimers **9** and **10**, along with trimers **11** and **12**. When the reaction was run with 0.5 equiv of CuI in MeCN at 23 °C, only trace amounts of dimers **9** and **10** were detected in the crude product; the major products isolated by flash chromatography were the trimers **11** (42%) and **12** (33%). The isolated yields of **11** and **12** were comparable to those of **6** and **7**.

**Scheme 4:**
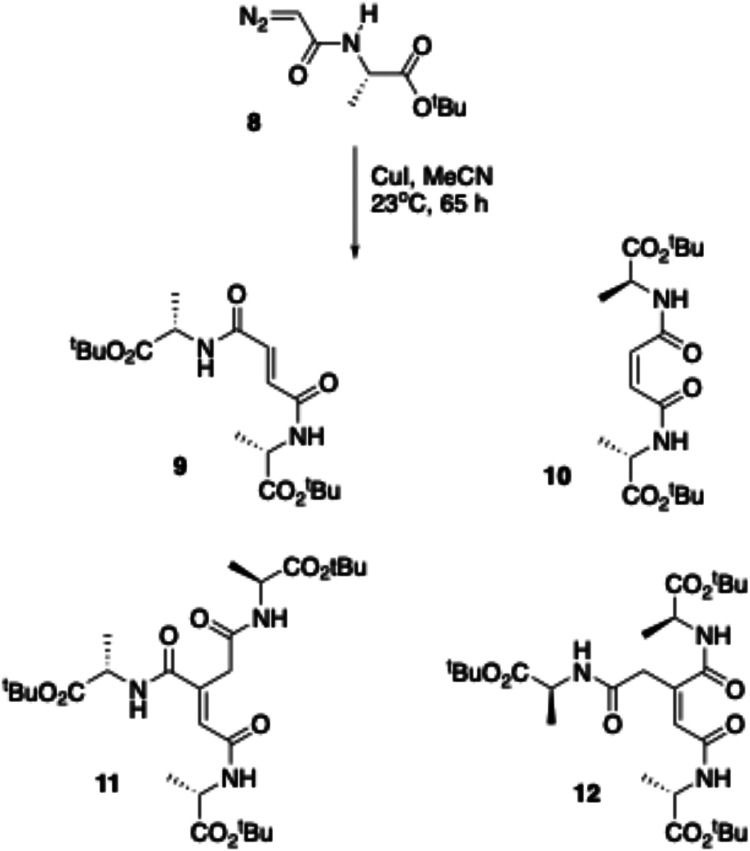
Dimerization (**9** and **10**) and Trimerization (**11** and **12**) Products Obtained from 8, an Alanine Diazopeptide

Like **6** and **7**, the trimers **11** and **12** are peptide derivatives of the (*E*) and (*Z*) isomers of aconitic acid, and they were identified as trimers from their molecular masses ascertained from their electrospray and high-resolution mass spectra. The structures of **11** and **12** were assigned on the basis of comparison of their ^1^H NMR spectra to the spectra of **6** and **7**. For trimer **11**, the alkene CH appears at 6.89 ppm, identifying it as a peptide derivative of (*E*)-aconitic acid, while for trimer **12**, the alkene CH appears at 6.21 ppm, identifying it as a peptide derivative of (*Z*)-aconitic acid.

The reaction conditions for producing **6** and **7** were optimized by varying the amount of CuI catalyst present. The best yields of **6** (46%) and **7** (28%) were obtained using 0.5 equiv of CuI. The use of lower percentages of CuI did produce **6** and **7**, but the formation of side products, primarily dimers similar to **9** and **10**, was noted and made isolation of the trimers more difficult. These dimers are either not present or present in small quantities when 0.5 equiv of CuI is used.

A notable feature of these reactions is that they yield both the (*E*) and (*Z*) isomers, with the (*E*) isomer being the major product. However, trimerizations like these have been reported previously for diazoacetates, and only the (*E*) isomers were isolated.^9–11^

A possible mechanism for the formation of these trimers is given in Scheme 5. It is known that diazocarbonyls (**13**) (like **4** and **8)** will react with CuI to lose N_2_ and generate Cu-carbene complexes (**14**).^19^ Addition of **14** to an unreacted diazocarbonyl **13** would yield the zwitterionic intermediate **15**, which can then lose another N_2_ to generate the dimer **16**, which could be either *cis* or *trans*. Conjugate addition of another **14** to **16** would yield another zwitterionic intermediate **17**, which could then reform the C=O, triggering a 1,2-hydride shift to produce the trimeric product **18**. Support for this mechansim comes from our observation that dimeric products are produced in these reactions, particularly when the amount of CuI is low. That increasing the concentration of CuI, which would increase the concentration of the Cu-carbene **14**, would be expected to increase the yield of the trimeric products over the dimeric products.

**Scheme 5:**
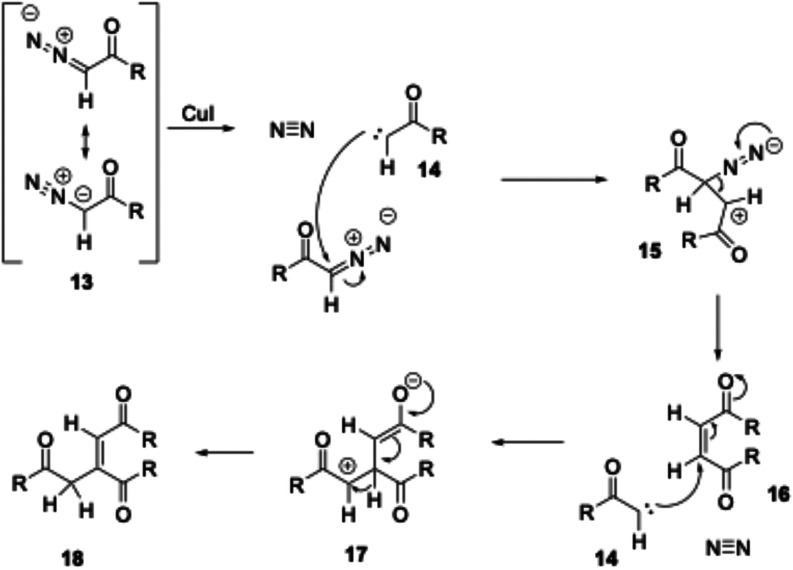
Possible Mechanism for the Formation of Trimeric Products^a^

a The proposed mechanism features the known addition of a Cu-carbene to the diazocarbonyl and also the conjugate addition of the Cu-carbene to the dimeric alkene product.

The ^1^H NMR spectra for **6** and **7** and for **11** and **12** show three amide NH protons. The chemical shifts for many of these NH protons are well above 7 ppm, indicating that they are deshielded. Given the close proximity of the functional groups in these molecules, a likely source of this deshielding could be intramolecular hydrogen bonding. To investigate this possibility, DMSO titrations^20–25^ of **6**, **7**, **11**, and **12** were undertaken.

In the DMSO titration experiment, the target molecule is dissolved in a solvent such as CDCl_3_ that is not an aggressive hydrogen bond acceptor. The ^1^H NMR spectrum is obtained, and the chemical shifts of the amide NH protons are recorded. To the CDCl_3_ solution is then added a small amount of *d*_6_-DMSO, a solvent that is an aggressive hydrogen bond acceptor. The ^1^H NMR spectrum is obtained again, and the new chemical shifts of the amide NH protons are recorded. The process of adding small volumes of *d*_6_-DMSO is continued until the percent DMSO is above 15%. If an amide NH proton is exposed to the solvent, then its chemical shift will undergo a large change (typically around 1 ppm or greater) as *d*_6_-DMSO is added. On the other hand, if an amide NH is already involved in an intramolecular hydrogen bond, then its chemical shift remains relatively constant. Thus, the behavior of the amide NH protons in the DMSO titration experiment can indicate the presence or absence of intramolecular hydrogen bonds.

DMSO titrations were performed on **6** and **7** and on **11** and **12**. The data from these experiments were plotted and are listed in Figure 1. Each compound has three different amide NH protons. In all four compounds, two of the three amide NH protons show little or no change in their chemical shifts, indicating that they are involved in intramolecular hydrogen bonds. In contrast, in all four compounds, there is one amide NH whose chemical shift changes significantly in the experiment, going up by 1 ppm or higher, indicating that this NH is exposed to the solvent.

**Figure 1:**
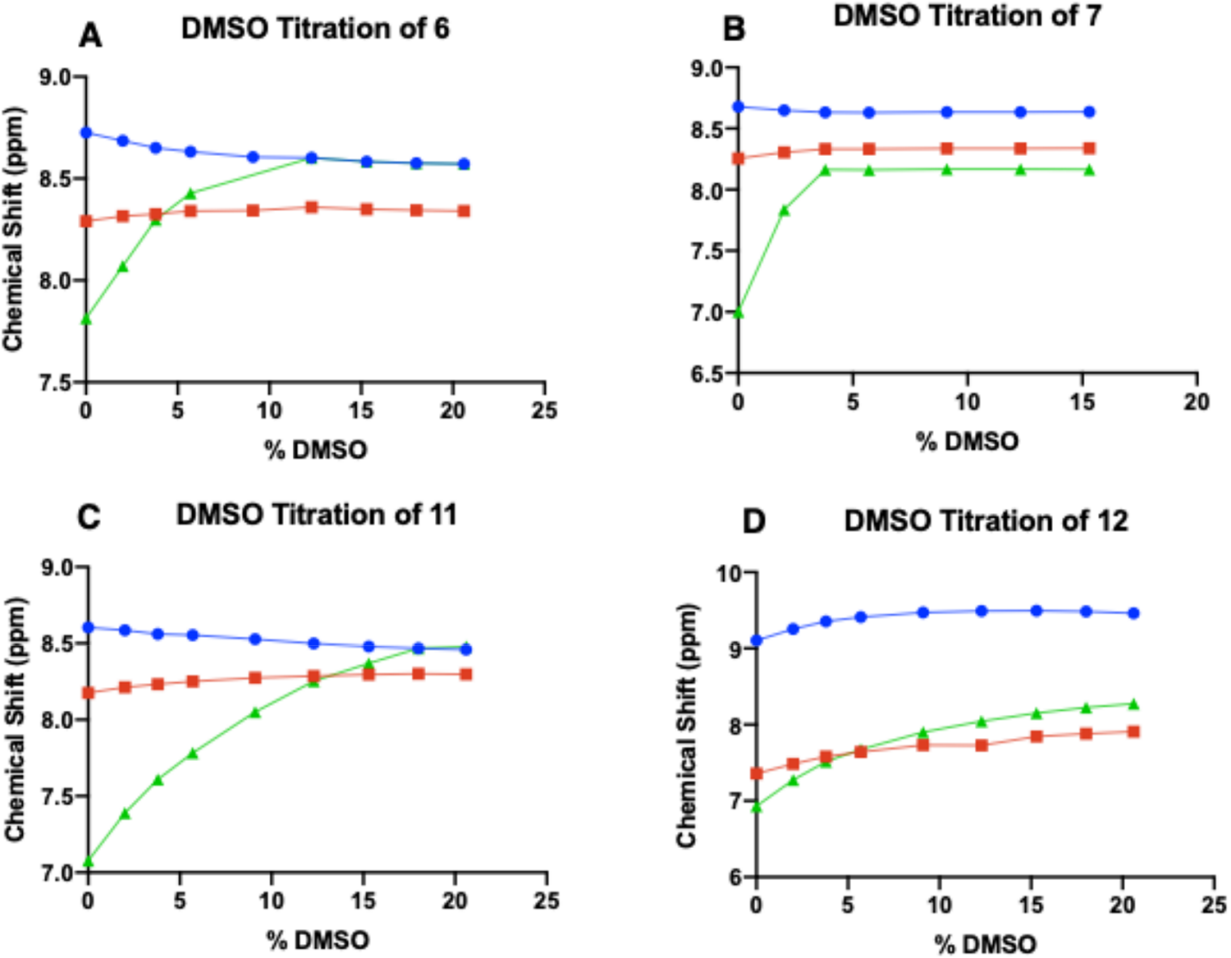
DMSO titration data for **6** (A), **7** (B), **11** (C), and **12** (D) showing the change in chemical shift for the three amide NH protons as *d*_6_-DMSO is added to a CDCl_3_ solution of each compound. The data shows that in each compound, two of the three amide NH protons undergo little to no change in their chemical shift as the *d*_6_-DMSO is added, indicating that these NH protons are involved in intramolecular hydrogen bonds.

The similar NMR spectra and DMSO titration results for **6** and **11**, and **7** and **12**, indicate that they have identical conformations. Accordingly, we only did calculations on **6** and **7** to determine their conformations and extrapolated the results to **10** and **11**. The conformational populations of **6** and **7** were preliminarily assessed in the MMFF94 force field. The low-energy conformations calculated for **6** and **7** are shown in Figure 2. For **6**, its conformation features two intramolecular hydrogen bonds that are parallel to each other, with the third NH positioned perpendicular to the two intramolecular hydrogen bonds. In contrast, for **7**, its conformation has the two intramolecular hydrogen bonds that are aligned roughly perpendicular to each other. With both **6** and **7**, the hydrogen bond labeled **x** in Figure 2 holds two of the amino acid residues in a β-sheet-like arrangement.

**Figure 2:**
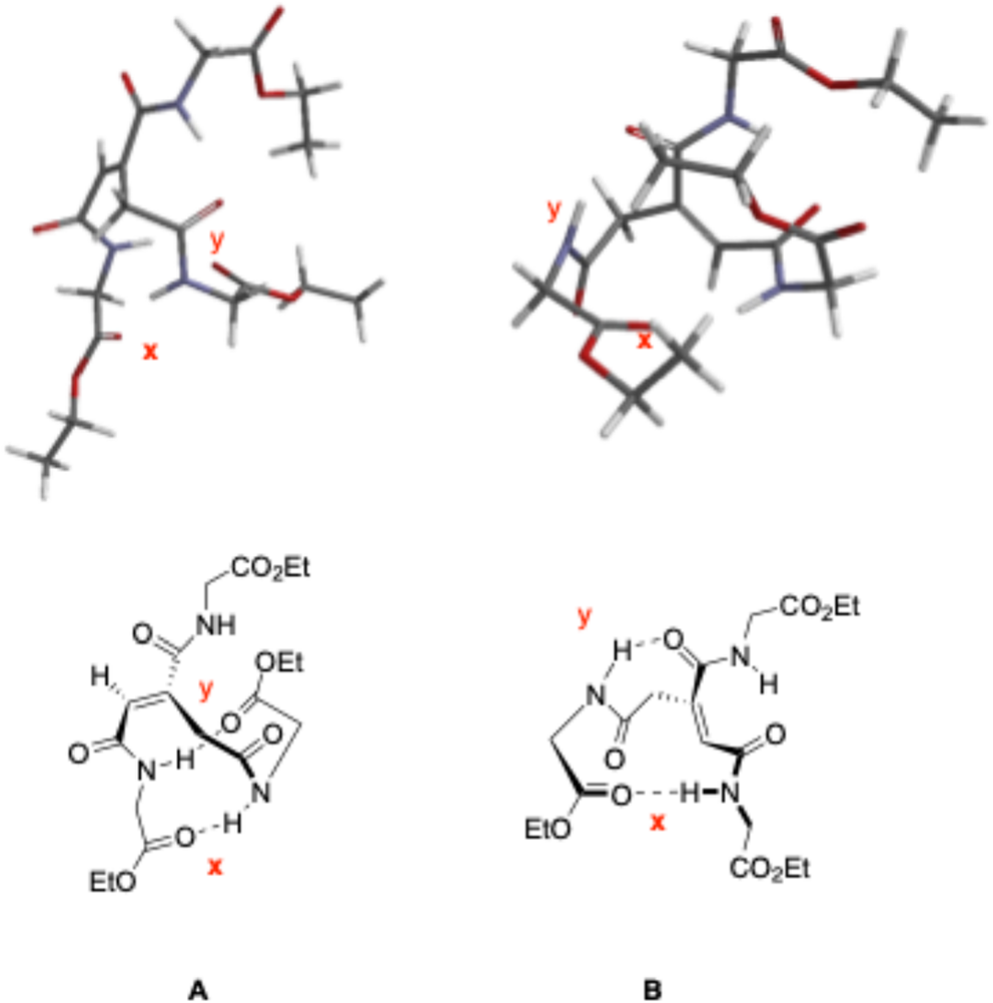
Calculated solution conformations of **6** (**A**) and **7** (**B**). Two of the three amide NH participate in intramolecular hydrogen bonds. The two intramolecular hydrogen bonds are labeled **x** and **y**. Trimers **11** and **12** adopt similar conformations.

Conformational searches on the **6** and **7** compounds were then investigated by using a TB-DFT/full DFT approach to yield a more accurate insight into the free energy landscape. The quantum chemistry calculations showed that **6** and **7** conformations gain 76.8% and 23.1% of the Boltzmann population, i.e., approximately 3:1 ratio, in full agreement with the experimental outcomes. Therefore, the quantum chemistry calculations evidenced that while **6** is represented by three conformers, with populations of 44.9%, 23.6%, and 8.3%, **7** is represented by only one conformer (Figure 3). Interestingly, all populated conformations of **6** and **7** compounds bear two hydrogen bonds involving amide groups, but always excluding the amide group directly bound to the alkenyl CH group (Figure 3).

**Figure 3:**
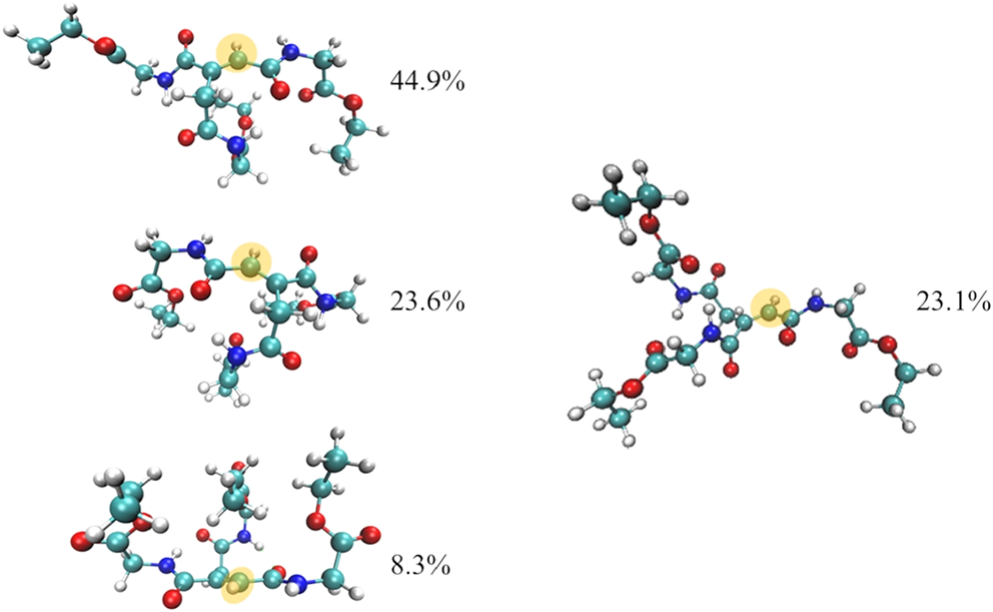
Ball-and-sticks representation of the most populated conformations of **6** (left) and **7** (right) compounds calculated by using the TB-DFT/full DFT approach. Hydrogen bonds are shown with dashed green lines. The alkenyl CH group is highlighted in yellow. The Boltzmann population percentages are also reported.

To test the validity of the calculated conformations of **6** and **7**, we next obtained the NOESY spectra of their alanine counterparts **11** and **12**. Given that the molecules are trimers, there are many areas of the spectrum where absorbances from the three amino acid residues overlap. However, there are two places of asymmetry in the trimers: the alkenyl CH and the allylic CH_2_ protons. Of particular interest was the proximity of these two sets of resonances to those of the three amide NH protons.

The NOESY data for **11** are given in Table 1. Of note here is that the NH that is not involved in an intramolecular hydrogen bond (7.26 ppm) is not in close proximity to the other two amide NH protons. The two NHs involved in intramolecular hydrogen bonds (8.63 and 8.21 ppm) are in close proximity to the unique allylic CH_2_ and the alkenyl CH. All of these close contacts are consistent with the calculated conformer for **6** (and **11**) shown in Figure 2A and in Figure 3, showing the amide group not involved in hydrogen bonds further apart from the allylic CH_2_.

**Table 1:** NOESY Cross-Peaks for Selected Resonances in **11**

		NOESY cross-peaks: δ (identity)		
peak (δ ppm)	peak identity	strong	moderate	weak
8.63^a^	NH	3.66 (CH_2_)	6.91 (=CH)	8.11 (NH)
		1.47 (^t^Bu)		4.48 (C_α_H)
8.21^a^	NH	3.66 (CH_2_)	4.47 (C_α_H)	
		1.39 (Ala CH_3_)		
7.26	NH		6.91 (=CH)	4.54 (C_α_H)
			1.42 (^t^Bu)	3.66 (CH_2_)
6.91	=CH	8.22 (NH)	8.62 (NH)	
3.66	CH_2_	8.63 (NH)	7.29 (NH)	6.91 (=CH)
		8.24 (NH)	1.45 (^t^Bu)	

a NH protons were involved in an intramolecular hydrogen bond.

The NOESY data for **12** are given in Table 2. The allylic CH_2_ here shows strong cross-peaks with both the NH involved in intramolecular hydrogen bonds, but no cross-peak to the other amide NH that is not hydrogen-bonded. The alkene CH shows strong cross-peaks with the allylic CH_2_ and the amide NH, not in an intramolecular hydrogen bond. The two NH that are involved in intramolecular hydrogen bonds show weak cross-peaks to the alkenyl CH. This pattern is consistent with the conformation for **7** (and **12**) shown in Figures 2 and 3.

**Table 2:** NOESY Cross-Peaks for Selected Resonances in **12**

		NOESY cross-peaks: δ (identity)		
peak (δ ppm)	peak identity	strong	moderate	weak
9.07^a^	NH	4.52 (C_α_H)	7.31 (NH)	6.20 (=CH)
		1.45 (^t^Bu)	3.22 (CH_2_)	4.48 (C_α_H)
7.31^a^	NH	3.22(CH_2_)	4.40 (C_α_H)	9.07 (NH)
		1.35 (Ala CH_3_)		
6.88	NH	6.20 (=CH)	4.50 (C_α_H)	
		1.38 (Ala CH_3_)		
6.20	=CH	6.86 (NH)	1.40 (Ala CH_3_)	7.30 (NH)
		3.23 (CH_2_)		9.07 (NH)
3.23	CH_2_	6.20 (=CH)		9.07 (NH)
		7.32 (NH)		

a NH protons involved in an intramolecular hydrogen bond.

In both isomers, two of the amino acid residues form a hydrogen bond and are positioned parallel to each other, as they might be found in a β-sheet. The third amino acid residue is located perpendicular to the two amino acid residues that hydrogen bond with each other. In one light, these species can be viewed as nascent peptide dendrimers that emanate from the central alkene core. Typically, peptide dendrimers have been generated from conformationally flexible core units, using polyamines or polyamino acids (like polylysine).^26–31^ Unlike polyamines or polyamino acids, the alkene core in these trimers limits the conformational freedom of the attached peptides and aligns the peptide chains to specific conformations. Such conformationally defined dendrimers might display interesting physical and biochemical properties.

## Experimental Section

### General

CuI was purchased from Acros Organics. MeCN, hexanes, EtOAc, CH_2_Cl_2_, and magnesium sulfate were purchased from Fisher Scientific. Deuterated solvents were purchased from Cambridge Isotope Laboratories, Inc., and silica gel for flash chromatography was purchased from Silicycle. NMR spectra were obtained on a Bruker Avance III 400 MHz instrument. Electrospray mass spectra were obtained on an LCQ APCI/electrospray LC MS-MS. Samples for mass spectral analysis were dissolved in MeOH (approximately 1 mg/mL) in borosilicate glass test tubes. Theoretical mass spectral isotope patterns were calculated using the online isotope pattern calculator provided on the Internet by the Swiss Federal Institute of Aquatic Science and Technology (Eawag).^32^ High-resolution mass spectra were obtained using an AB Sciex QSTAR Elite, employing an orthogonal time-of-flight (TOF) analyzer, at the University of Connecticut Laboratory of Mass Spectrometry and Omics Analysis. Initial calculations were performed by using Spartan 24.1.3.1. A random search of possible conformations was employed. The force field MMFF94 was used to find the lowest-energy conformer. Structural assignments were made with additional information from gNOESY experiments.

### Preparation of **6** and **7**

To a mixture under N_2_ of 231 mg of **4** (1.35 mmol, 1.0 equiv) and 116 mg of CuI (0.609 mmol, 0.45 equiv) was added 4.0 mL of degassed CH_3_CN. The solution was stirred at 23 °C for 24 h. The solvent was evaporated, and the crude product was subjected to flash chromatography. The eluent 4:1 EtOAc/hexane provided 89 mg (46%) of **6** as a white solid. Changing the eluent to 9:1 EtOAc/MeOH then afforded 55 mg (28%) of **7** as a white solid:

**6**: TLC *R*_f_ = 0.46 (4:1 ethyl acetate/hexane); ^1^H NMR (400 MHz, CDCl_3_) δ 8.72 (1H, t, *J* = 4.9 Hz), 8.30 (1H, t, *J* = 5.3 Hz), 7.97 (1H, m), 7.08 (1H, s), 4.25–4.15 (6H, m), 4.09 (2H, d, *J* = 5.3 Hz), 4.05 (2H, d, *J*= 5.3. Hz), 3.99 (2H, d, *J* = 5.6 Hz), 3.74 (2H, s), 1.32–1.21 (9H, m); ^13^C NMR­{1H} (100 MHz, CDCl_3_) δ 171.3, 169.6, 169.4, 169.3, 167.7, 166.2, 139.6, 129.1, 61.6, 61.5, 61.4, 42.2, 41.6, 41.5, 36.3, 14.1, 14.1, 14.0; MS (ESI) *m*/*z*: [M + Na]^+^ Calcd for for C_18_H_27_N_3_O_9_Na 452.16; Found: 452.34. HRMS: [M + H]^+^ Calcd for C_18_H_28_N_3_O_9_: 430.1820; Found: 430.1793.

**7**: TLC *R*_f_ = 0.26 (4:1 ethyl acetate/hexane); ^1^H NMR (400 MHz, CDCl_3_) δ 9.25 (1H, t, *J* = 4.8 Hz), 7.33 (1H, t, *J* = 5.4 Hz), 6.91 (1H, t, *J* = 5.3 Hz), 6.33 (1H, s), 4.28–4.15 (6H, m), 4.11 (2H, d, *J* = 5.5 Hz), 4.07 (2H, d, *J* = 5.2 Hz), 3.99 (2H, d, *J* = 5.6 Hz), 3.33 (2H, s), 1.33–1.22 (9H, m); ^13^C NMR­{1H} (100 MHz, CDCl_3_) δ 170.4, 170.0, 169.8, 169.6, 167.6, 165.2, 140.2, 127.8, 61.6, 61.5, 61.4, 42.9, 41.8, 41.6, 41.5, 14.1, 14.1, 14.0. MS (ESI) *m*/*z*: [M + Na]^+^ Calcd for for C_18_H_27_N_3_O_9_Na 452.16; Found: 452.40. HRMS: [M + H]^+^ Calcd for C_18_H_28_N_3_O_9_: 430.1820; Found: 430.1786.

### Preparation of **11** and **12**

To a mixture under N_2_ of 58 mg of **8** (0.272 mmol, 1.0 equiv) and 25 mg of CuI (0.131 mmol, 0.5 equiv) was added 1.0 mL of degassed CH_3_CN. The solution was stirred at 23 °C for 24 h. The solvent was evaporated and the crude product was subjected to flash chromatography with a succession of eluents: 3:1 hexane/EtOAc, 2:1 hexane/EtOAc, 3:2 hexane/EtOAc and finally 1:4 hexane/EtOAc. The eluent 2:1 hexane/EtOAc provided 21 mg (42%) of **11** as a white solid. The eluent 1:4 hexane/EtOAc then afforded 16 mg (33%) of **12** as a white solid:

**11**: TLC *R*_f_ = 0.39 (50% hexanes/50% EtOAc); ^1^H NMR (400 MHz, CDCl_3_) δ 8.63 (1H, d, *J* = 6.9 Hz), 8.20 (1H, d, *J* = 7.8 Hz), 7.26 (1H, d, *J* = 7.4 Hz), 4.58–4.36 (3H, m), 3.64 (2H, dd, *J* = 12.4 and 25.1 Hz), 1.49–1.42 (30H, m), 1.40 (3H, d, *J* = 7.2 Hz), 1.35 (3H, d, *J* = 7.2 Hz); ^13^C NMR­{1H} (100 MHz, CDCl_3_) δ 172.0, 171.7, 171.6, 170.2, 166.8, 165.3, 139.4, 128.9, 82.3, 81.8, 81.7, 49.6, 49.0, 48.3, 36.6, 27.93, 27.91, 18.0, 17.9. [overlapping peaks at 27.9 and 17.9 ppm]; MS (ESI) *m*/*z*: [M + Na]^+^ Calcd for for C_27_H_45_N_3_O_9_Na: 578.30; found: 578.61; HRMS: [M + Na]^+^ Calcd for C_27_H_45_N_3_O_9_Na: 578.3048; Found: 578.2983.

**12**: TLC *R*_f_ = 0.18 (50% hexanes/50% EtOAc); ^1^H NMR (400 MHz, CDCl_3_) δ 9.13 (1H, br s), 7.39 (1H, d, *J* = 6.7 Hz), 7.03 (1H, br s), 6.21 (1H, s), 4.58–4.44 (2H, m), 4.41 (1H, pentet, J-7.3 Hz), 3.25 (2H, dd, *J* = 13.3 and 24.3 Hz), 1.49–1.42 (27H, m); ^13^C NMR­{1H} (100 MHz, CDCl_3_) δ 172.33, 172.29, 171.7, 169.1, 166.9, 163.9, 140.5, 127.1, 82.1, 82.0, 81.8, 49.3, 49.0, 48.9, 43.4, 28.0, 27.9, 18.4, 17.7, 17.4 [overlapping peaks at 27.9 ppm]; MS (ESI) *m*/*z*: [M + Na]^+^ Calcd for for C_27_H_45_N_3_O_9_Na: 578.30; found: 578.46; HRMS: [M + Na]^+^ Calcd for C_27_H_45_N_3_O_9_Na: 578.3048; Found: 578.3008.

### Quantum Chemistry Calculations

The most representative conformations of **6** and **7** (vide infra) were calculated using a combined tight-binding DFT/full DFT approach. The conformational sampling was carried out using the Conformer-Rotamer Ensemble Sampling Tool (CREST), an application software based on the xTB methods.^33^ The CREST calculation was executed by applying the default setup for the iMTD-GC workflow^34^ and the *alpb* option to model the acetonitrile solvation. The conformational ensembles were then clusterized in the VMD workspace by using the Clustering Tool plugin.^35^ The representative structures of the most populated clusters (>90% of all population) were then optimized by using the Gaussian 16 programs package^36^ at the ωB97XD/6-311++G** level of theory that was shown to produce reliable energy landscape insights in peptide-containing systems.^37,38^

## Supplementary Material



## Data Availability

The data underlying this study are available in the published article and its Supporting Information.

## Notes

Spectral data for compounds **6**, **7**, **9**, **10**, **11**, and **12** and the procedure for making **9** and **10** (PDF)
