# Supplementary material for: Copper-Mediated Trimerization of Diazopeptides Generates Peptide Derivatives of Cis- and Trans-Aconitic Acid That Adopt Folded Structures
Source: J Org Chem. 2026 Jun 1;91(23):8045–51. doi: 10.1021/acs.joc.5c02991 (PMC13270505; doi:10.1021/acs.joc.5c02991)
Supplement: Supplementary file 1 [file jo5c02991_si_001.pdf]

Supporting Information

Copper Mediated Trimerization of Diazopeptides Generates Peptide Derivatives of Cis- and Trans-Aconitic Acid That Adopt Folded Structures

Timothy P. Curran,[a]* Lilly L. Pubillones,[a] Alessandro Marrone,[b] Iogann Tolbatov,[c] and Sara C. Ingrey[a]

[a]Department of Chemistry, Trinity College, 300 Summit Street, Hartford, CT 06106 USA
[b]Dipartimento di Farmacia, Università degli Studi "G. D'Annunzio" Chieti-Pescara, Via dei Vestini 31, Chieti, 66100, Italy
[c]Department of Chemical, Physical, Mathematical and Natural Sciences, University of Sassari, Sassari, 07100, Italy

Email: timothy.curran@trincoll.edu

Table of Contents

Page **Contents**

Page	Contents
S3	Procedure for making **9**, **10**, **11** and **12**
S5	^{1}H NMR of **6**
S6	^{13}C (^{1}H) NMR of **6**
S7	ESI-MS of **6**
S8	HRMS of **6**
S9	^{1}H NMR of **7**
S10	^{13}C (^{1}H) NMR of **7**
S11	ESI-MS of **7**
S12	HRMS of **7**
S13	^{1}H NMR of **9**
S14	^{13}C (^{1}H) NMR of **9**
S15	ESI-MS of **9**
S16	HRMS of **9**
S17	^{1}H NMR of **10**
S18	^{13}C (^{1}H) NMR of **10**
S19	ESI-MS of **10**
S20	HRMS of **10**
S21	^{1}H NMR of **11**
S22	^{13}C (^{1}H) NMR of **11**
S23	ESI-MS of **11**
S24	HRMS of **11**
S25	gNOESY of **11**
S26	^{1}H NMR of **12**
S27	^{13}C (^{1}H) NMR of **12**
S28	ESI-MS of **12**
S29	HRMS of **12**
S30	gNOESY of **12**
S31	Note S1: Z-matrices of **6**
S40	Note S1: Z-matrices of **7**
S53	Table S1: Calculated Gibbs free energies for **6** and **7**
S54	Full gaussian 16 reference

*Reaction of **8** with 10 % CuI; Formation and Isolation of **9, 10, 11** and **12***

To a round-bottom flask containing 231 mg (1.08 mmol, 1.0 equiv.) of **8**[1] and 23 mg (0.12 mmol, 0.1 equiv.) of CuI under an atmosphere of N_2 was added 4.0 mL of degassed MeCN. The resulting yellow solution stirred under N_2. The yellow color was lost after 45 min. After stirring for 65 h the solvents were evaporated. The brown solid that remained was subjected to automated flash chromatography using the hexanes-ethyl acetate gradient. The first set of fractions contained a mixture of **9** and **11**, while the second set of fractions contained a mixture of **10** and **12**.

The mixture of **9** and **11** was subjected to a subsequent manual flash chromatography using a 2:1 hexanes/ethyl acetate as the eluant. This solvent eluted pure **9**. Evaporation of the appropriate fractions yielded 35 mg (18 %) of pure **9**. The eluant was changed to 1:2 hexanes/ethyl acetate and pure **11** was obtained. Evaporation of the appropriate fractions yielded 22 mg (11 %) of pure **11**.

The mixture of **10** and **12** was subjected to a subsequent manual flash chromatography using 3:2 hexanes/ethyl acetate as the eluant. This solvent eluted pure **10**. Evaporation of the appropriate fractions yielded 10 mg (5 %) of pure **5**. The eluant was changed to 1:4 hexanes/ethyl acetate and pure **12** was obtained. Evaporation of the appropriate fractions yielded 18 mg (9 %) of pure **12**.

9: TLC R_f=0.50 (50% hexanes/50% EtOAc); ^{1}H NMR (400 MHz, $CDCl_3$) δ 7.10 (1H, d, J=7.5 Hz), 7.04 (1H, s), 4.62 (1H, pentet, J=7.4 Hz), 1.47 (9H, s), 1.41 (3H, d, J=7.2 Hz); ^{13}C NMR{1H} (100 MHz, $CDCl_3$) δ 172.1, 163.6, 133.1, 82.2, 48.9, 28.0, 18.5. ESI-MS M + Na ion pattern calculated for $C_{18}H_{30}N_2O_6Na$: 393 (100), 394 (20.3), 395 (3.3); found: 393 (100), 394 (17.8), 395 (1.9); HRMS: $[M+H]^+$ Calcd. for $C_{18}H_{31}N_2O_6$: 371.2177; Found: 371.2162.

10: TLC R_f=0.27 (50% hexanes/50% EtOAc); ^{1}H NMR (400 MHz, $CDCl_3$) δ 8.49 (1H, d, J=7.0 Hz), 6.13 (1H, s), 4.52 (1H, pentet, J=7.2 Hz), 1.47 (9H, s), 1.43 (3H, d, J=7.2 Hz); ^{13}C NMR{1H} (100 MHz, $CDCl_3$) δ 171.9, 163.9, 132.5, 82.1, 49.1, 28.0, 18.3; ESI-MS M + Na ion pattern calculated for $C_{18}H_{30}N_2O_6Na$: 393 (100), 394 (20.3), 395 (3.3); found: 393 (100), 394 (17.0), 395 (3.6); HRMS: $[M+H]^+$ Calcd. for $C_{27}H_{46}N_3O_9$: 556.3229; Found: 556.3208.

Discussion:

Electrospray mass spectrometry and high-resolution mass spectrometry identified **9** and **10** as the dimers. Dimer **9** is a peptide derivative of fumaric acid, while dimer **10** is a peptide derivative of maleic acid. Their specific identities were determined from the presence of the alkene CH peaks in their ^{1}H NMR spectra. For fumaric acid and dimethylfumarate, the two alkene CH appear at 6.65 and 6.86 ppm,[2] while for maleic acid and dimethylmaleate the two alkene CH appear at 6.29 and 6.20 ppm.[2] These differences in chemical shift can be used to identify derivatives of fumaric acid from similar derivatives of maleic acid. Derivatives of

fumaric acid have the alkene CH located around 6.8 ppm, while for derivatives of maleic acid the alkene CH is located around 6.3 ppm. With dimer **9** the alkene CH peaks appear at 7.04 ppm, identifying it as a peptide derivative of fumaric acid. With dimer **10** the alkene CH peaks appear at 6.13 ppm, identifying it is a peptide derivative of maleic acid.

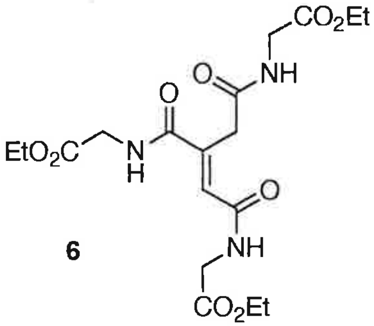

6

^{1}H NMR ($CDCl_3$)
400 MHz

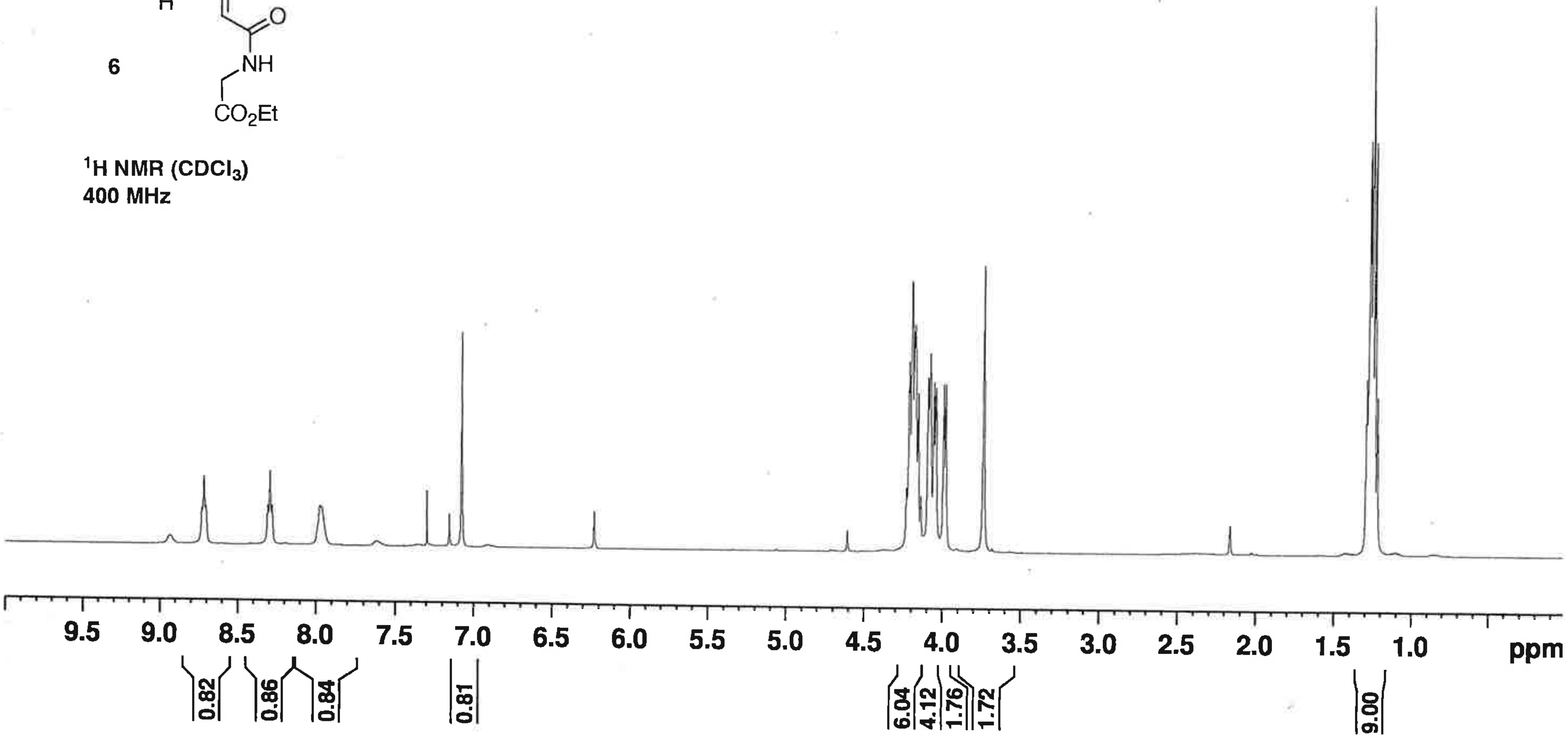

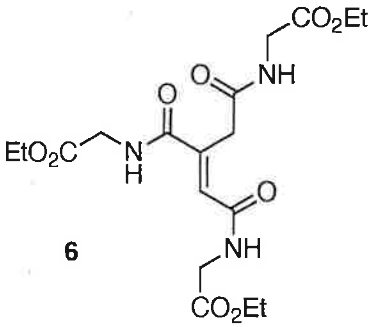

6

^{13}C (^{1}H) NMR ($CDCl_3$)
100 MHz

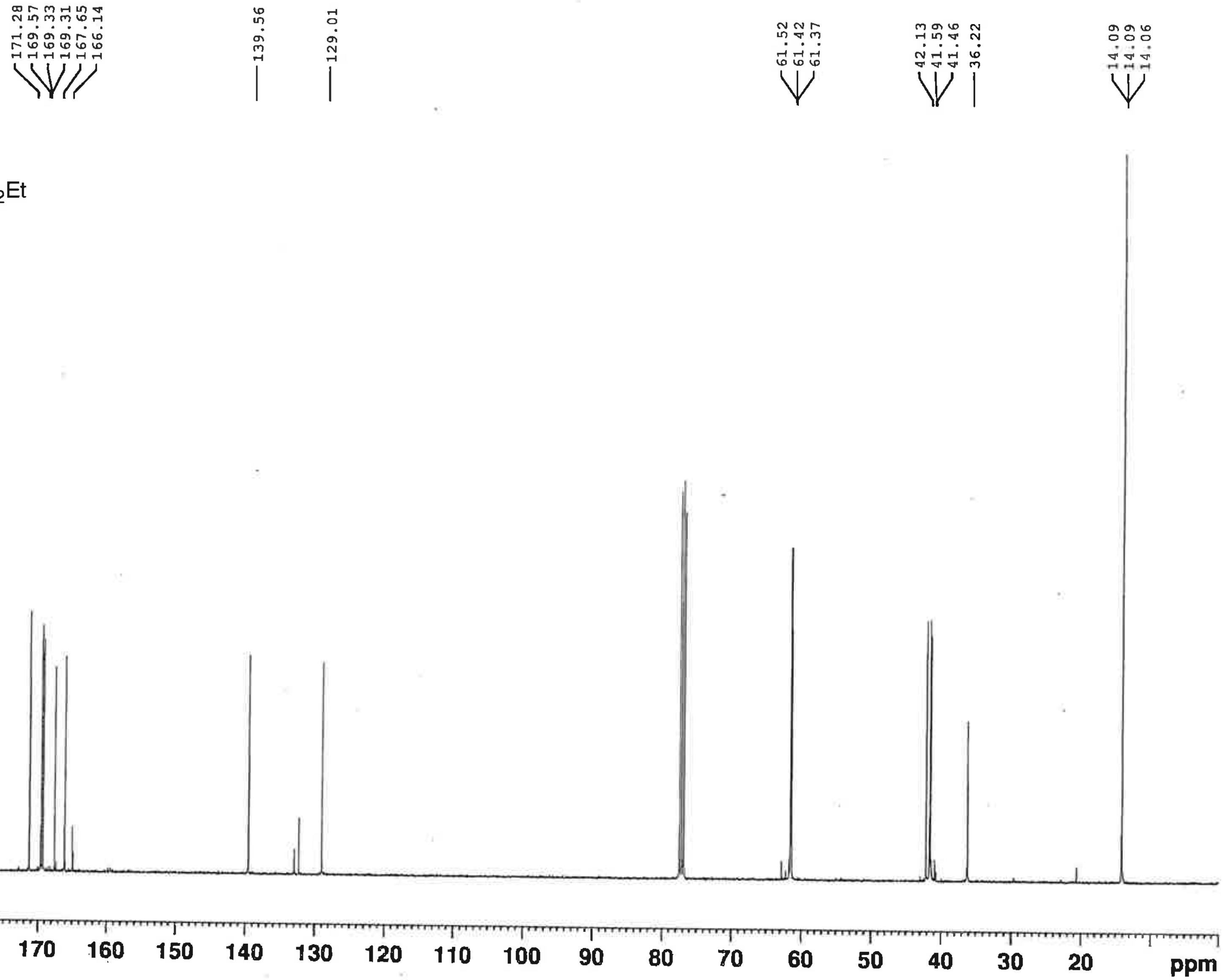

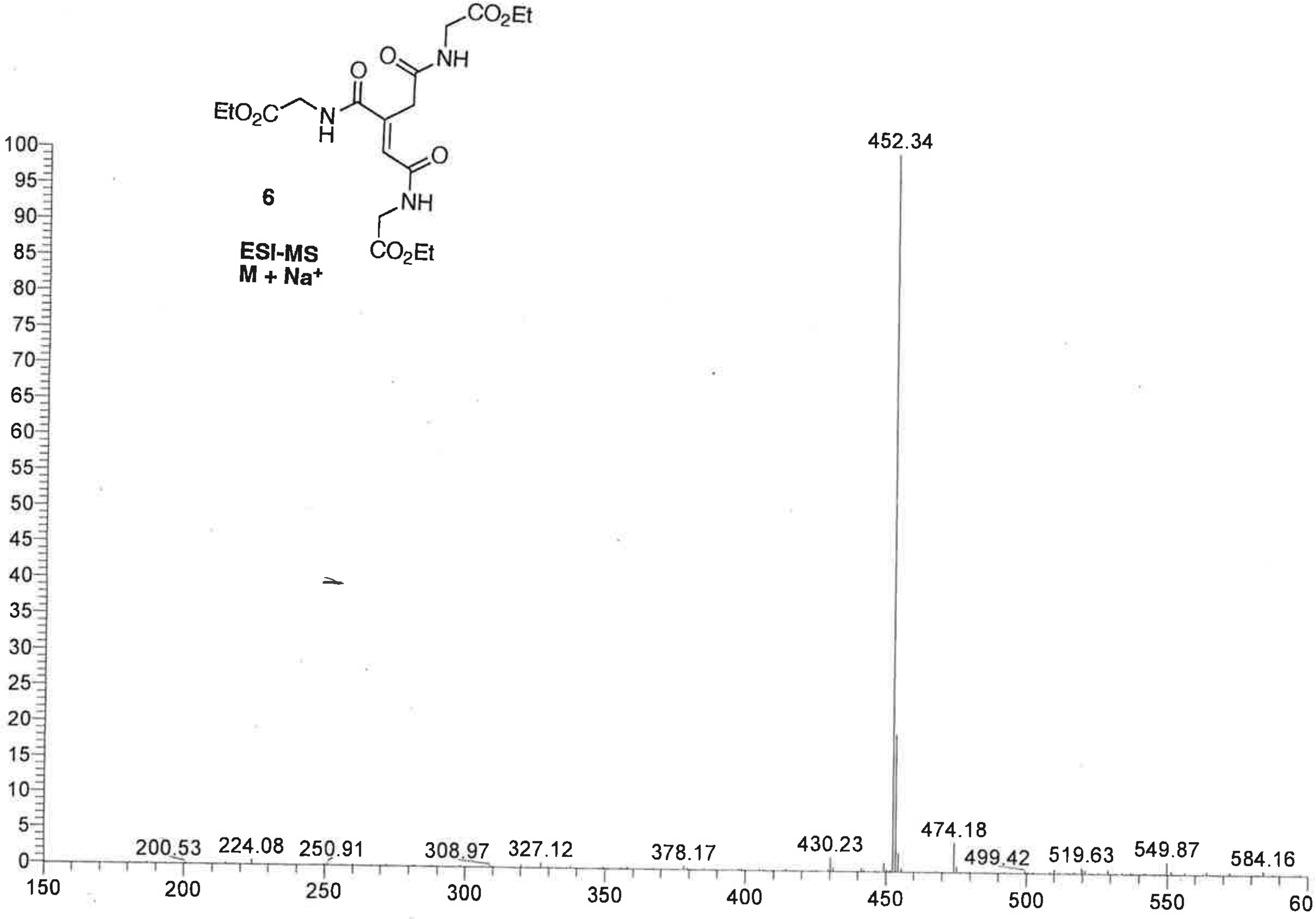
CO_2Et
O
NH
O
EtO_2C
N
H
O
NH
CO_2Et
6
ESI-MS
M + Na+
452.34
200.53
224.08
250.91
308.97
327.12
378.17
430.23
474.18
499.42
519.63
549.87
584.16
100
95
90
85
80
75
70
65
60
55
50
45
40
35
30
25
20
15
10
5
0
150
200
250
300
350
400
450
500
550
60

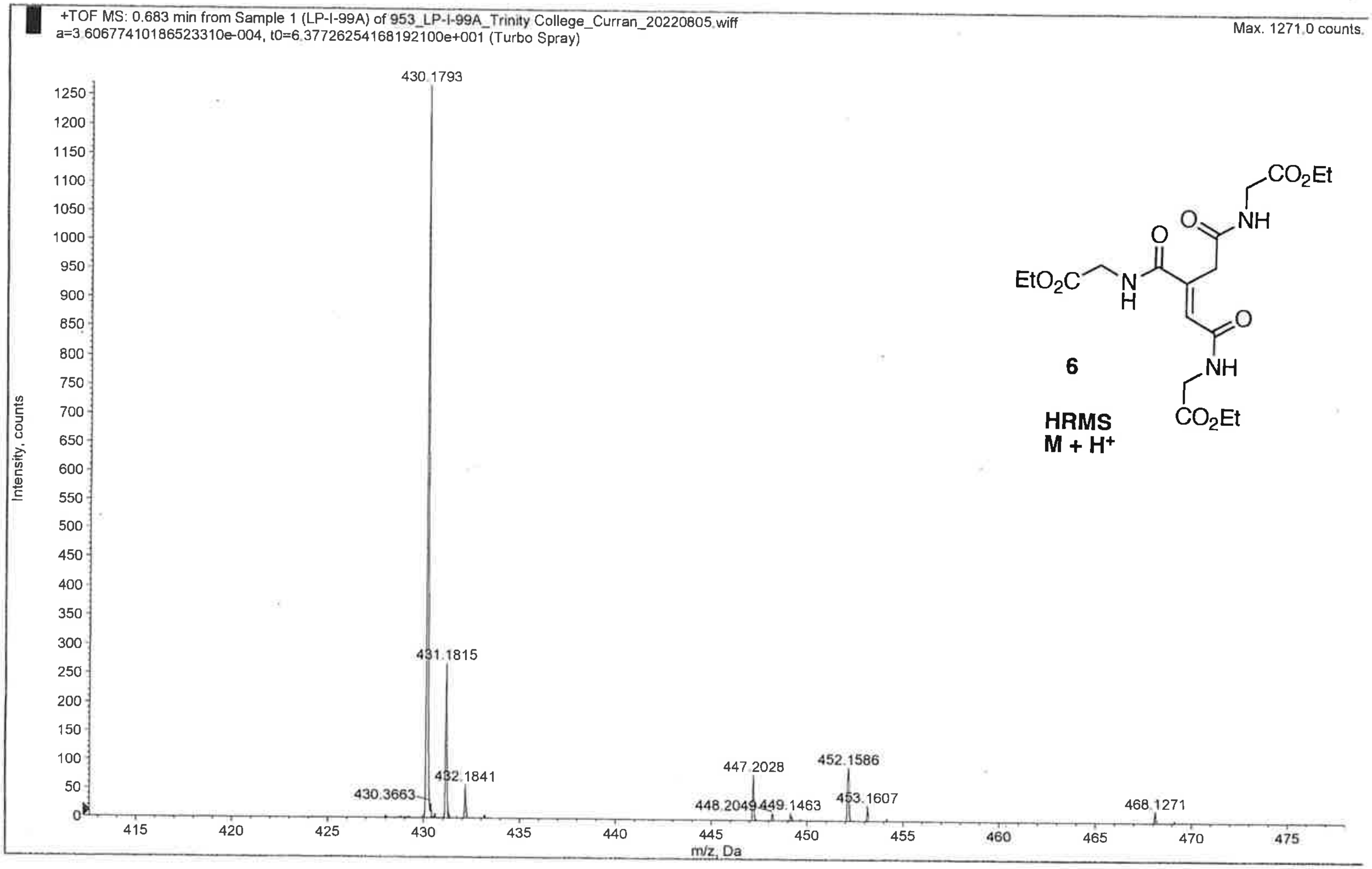
+TOF MS: 0.683 min from Sample 1 (LP-I-99A) of 953_LP-I-99A_Trinity College_Curran_20220805.wiff
a=3.60677410186523310e-004, t0=6.37726254168192100e+001 (Turbo Spray)
Max. 1271.0 counts.
Intensity, counts
0
50
100
150
200
250
300
350
400
450
500
550
600
650
700
750
800
850
900
950
1000
1050
1100
1150
1200
1250
430.1793
431.1815
432.1841
430.3663
447.2028
448.2049
449.1463
452.1586
453.1607
468.1271
415
420
425
430
435
440
445
450
455
460
465
470
475
m/z, Da
CO2Et
NH
O
O
EtO2C
N
H
O
NH
CO2Et

6

HRMS
$M + H^+$

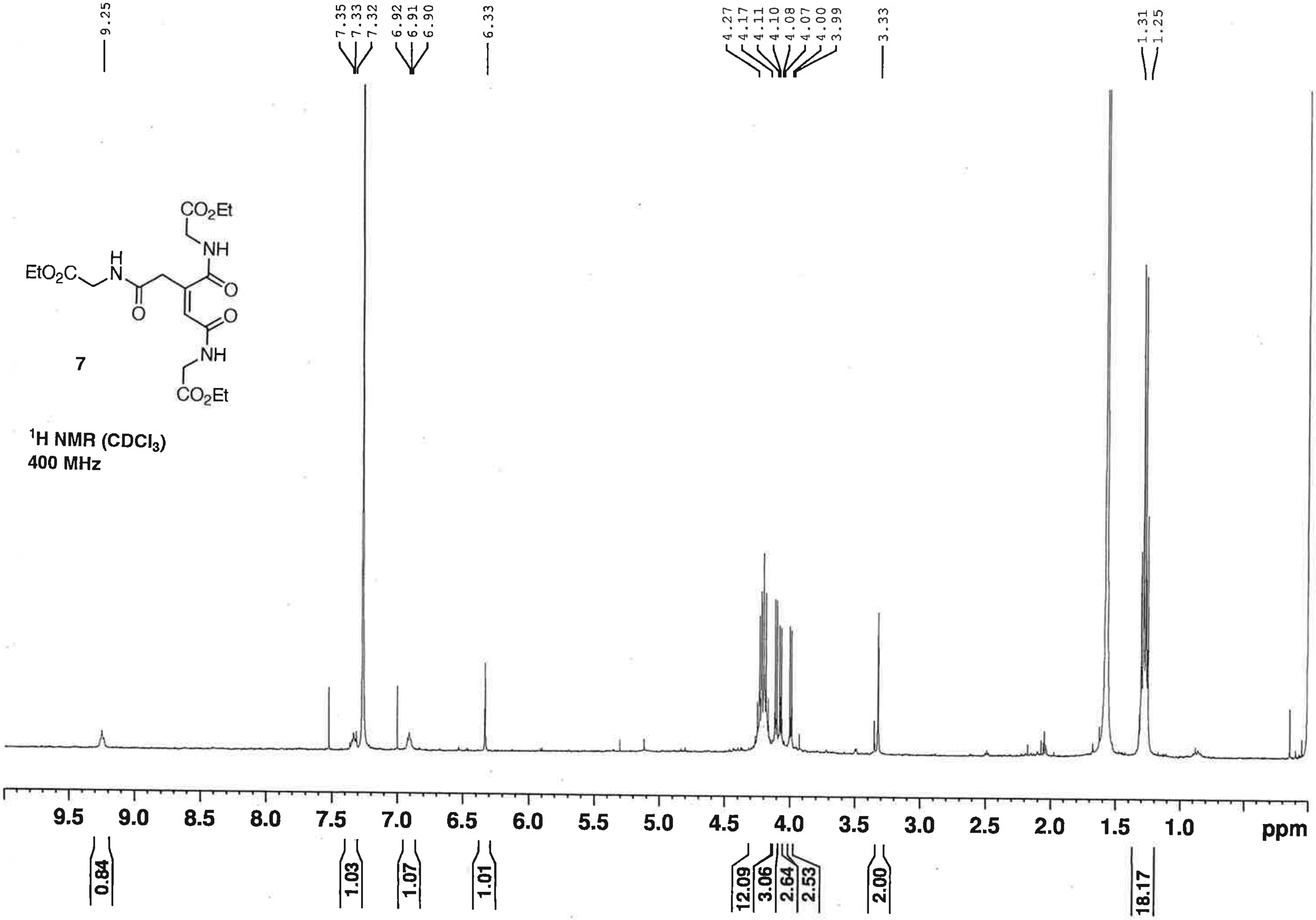

9.25
7.35
7.33
7.32
6.92
6.91
6.90
6.33
4.27
4.17
4.11
4.10
4.08
4.07
4.00
3.99
3.33
1.31
1.25
CO_2Et
NH
EtO_2C
H
N
O
O
O
NH
CO_2Et
7
^{1}H NMR ($CDCl_3$)
400 MHz
9.5
9.0
8.5
8.0
7.5
7.0
6.5
6.0
5.5
5.0
4.5
4.0
3.5
3.0
2.5
2.0
1.5
1.0
ppm
0.84
1.03
1.07
1.01
12.09
3.06
2.64
2.53
2.00
18.17

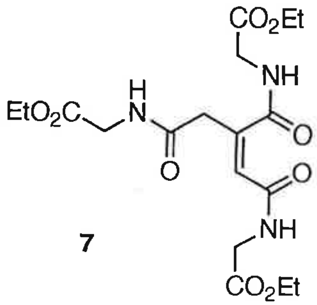

7

^{13}C (^{1}H) NMR ($CDCl_3$)
100 MHz

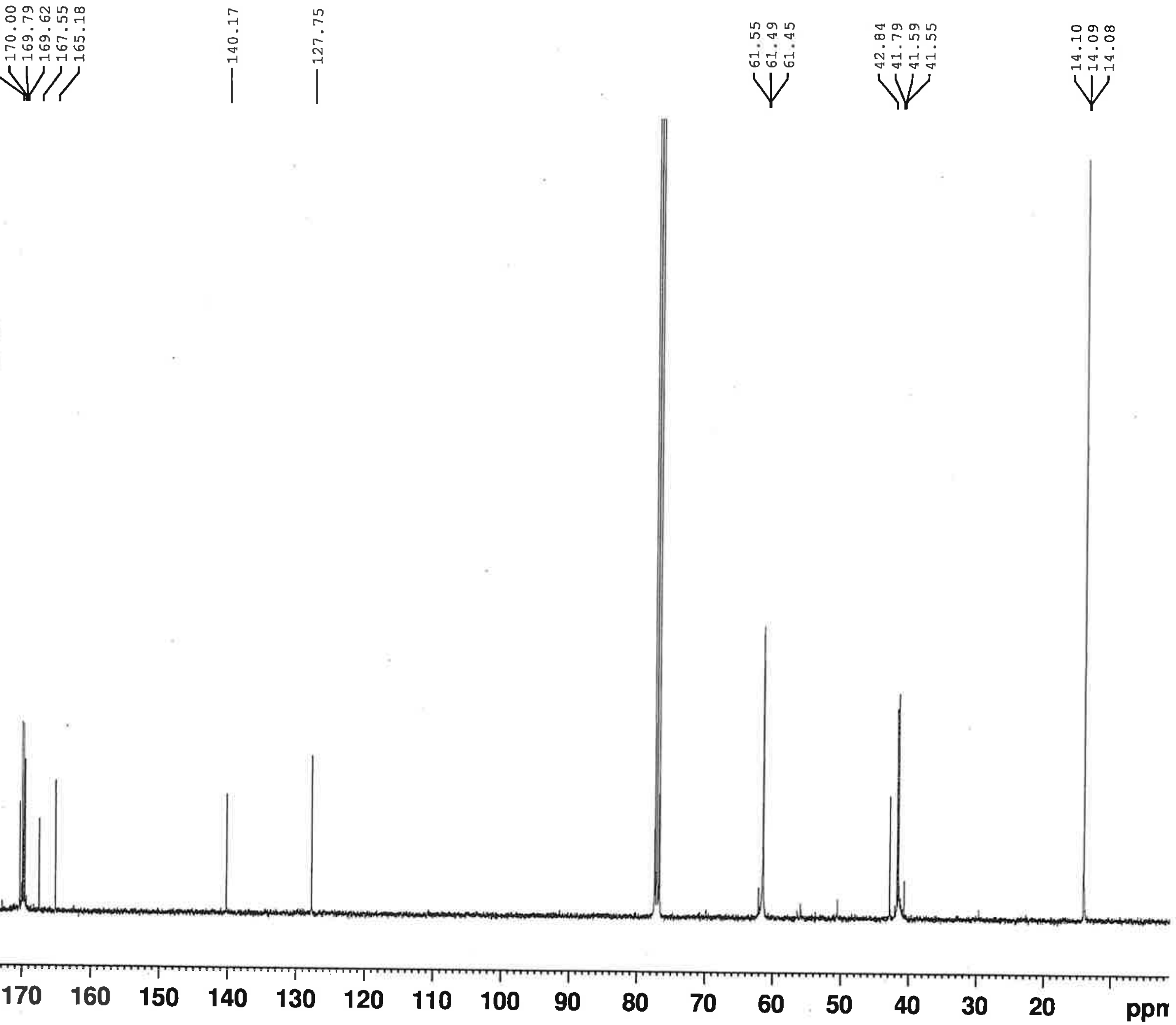

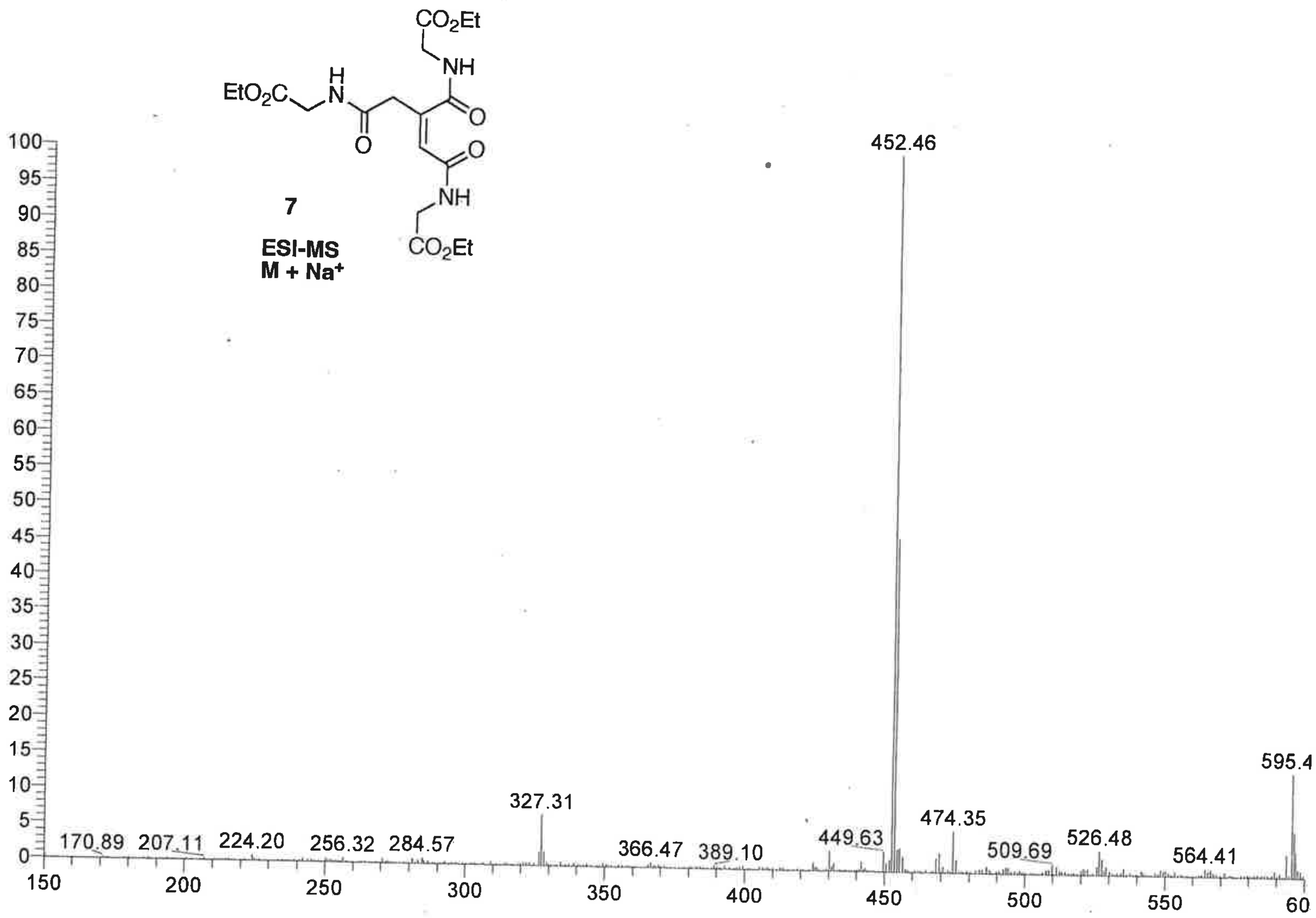
CO2Et
NH
EtO2C
H
N
O
O
O
NH
CO2Et
7
ESI-MS
M + Na+
452.46
595.4
327.31
474.35
449.63
526.48
509.69
564.41
366.47
389.10
170.89
207.11
224.20
256.32
284.57
100
95
90
85
80
75
70
65
60
55
50
45
40
35
30
25
20
15
10
5
0
150
200
250
300
350
400
450
500
550
60

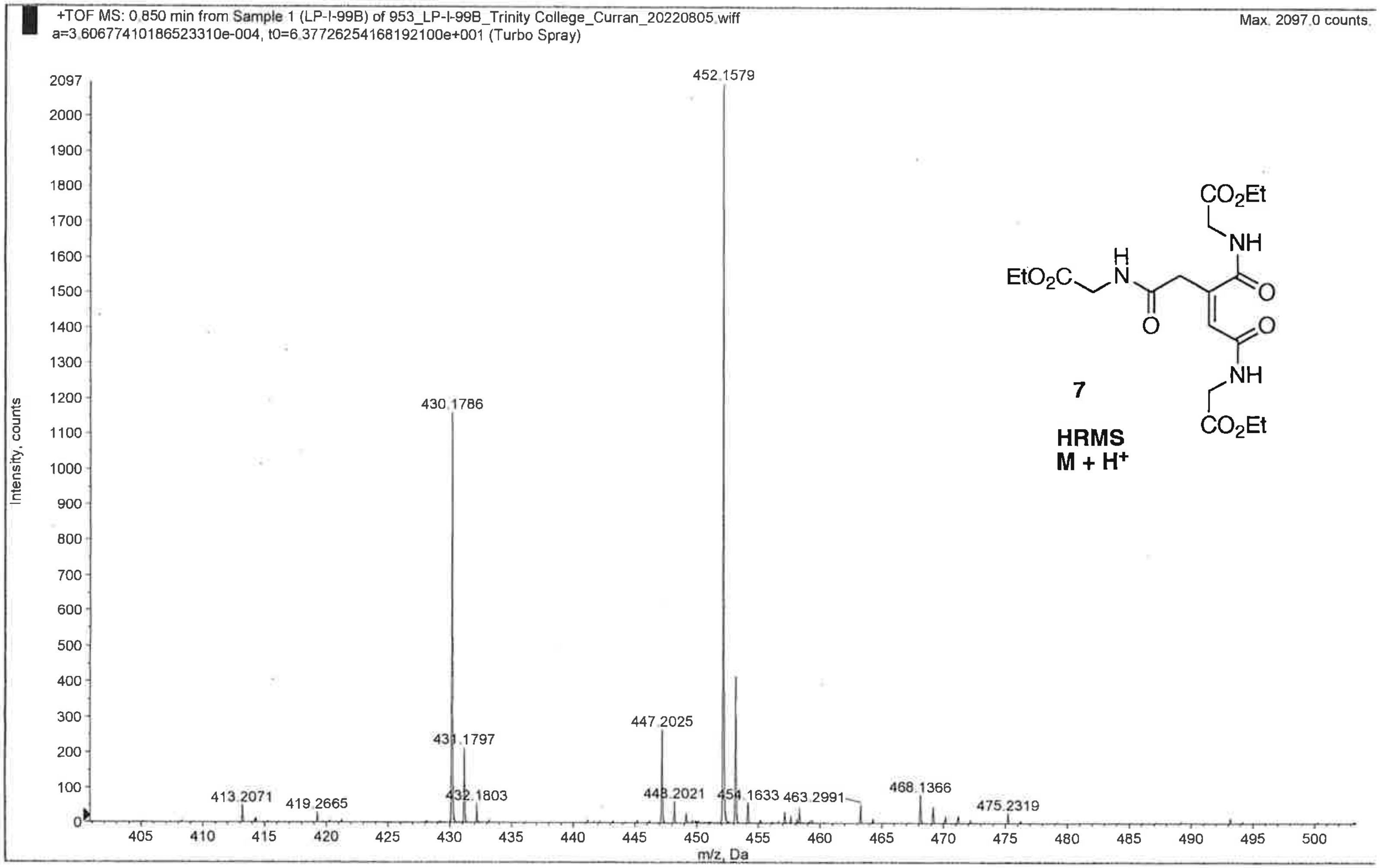
+TOF MS: 0.850 min from Sample 1 (LP-I-99B) of 953_LP-I-99B_Trinity College_Curran_20220805.wiff
a=3.60677410186523310e-004, t0=6.37726254168192100e+001 (Turbo Spray)
Max. 2097.0 counts.
Intensity, counts
m/z, Da
452.1579
430.1786
447.2025
431.1797
432.1803
413.2071
419.2665
448.2021
454.1633
463.2991
468.1366
475.2319
CO_2Et
NH
H
N
EtO_2C
O
O
O
NH
CO_2Et
7
HRMS
M + H+

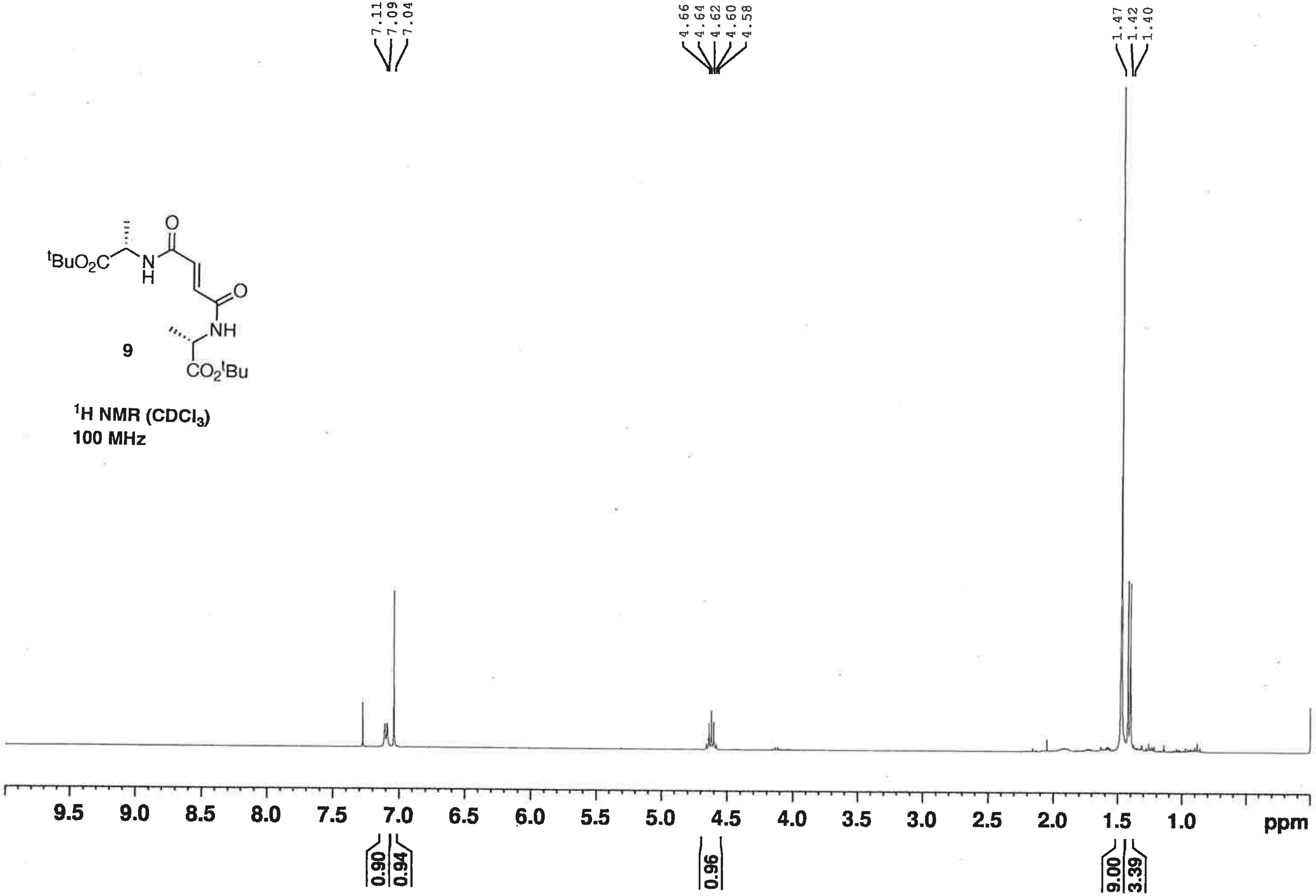

9

^{1}H NMR ($CDCl_3$)
100 MHz

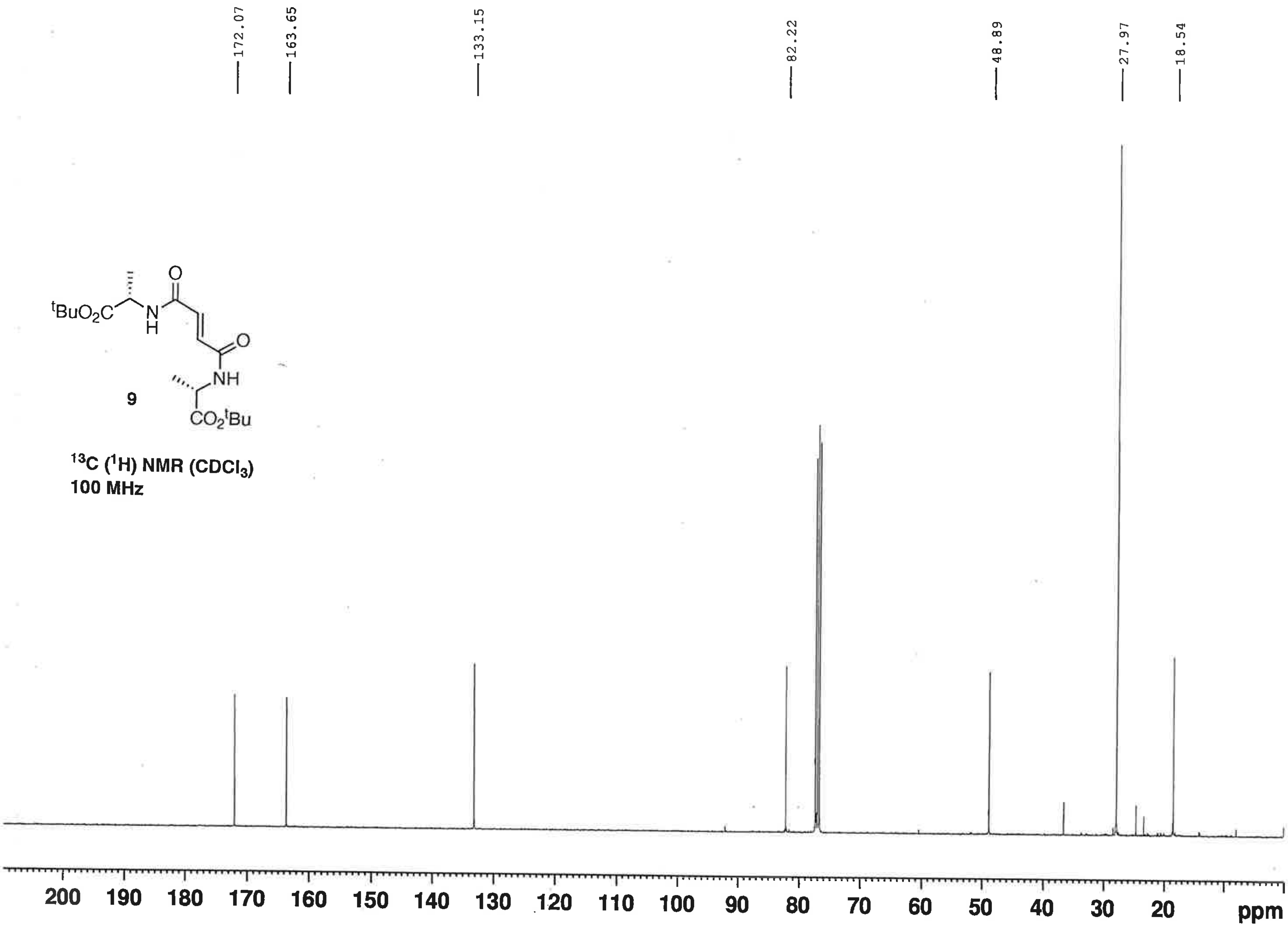
172.07
163.65
133.15
82.22
48.89
27.97
18.54
tBuO2C
N
H
O
O
NH
CO2tBu
9
13C (1H) NMR (CDCl3)
100 MHz
200 190 180 170 160 150 140 130 120 110 100 90 80 70 60 50 40 30 20
ppm

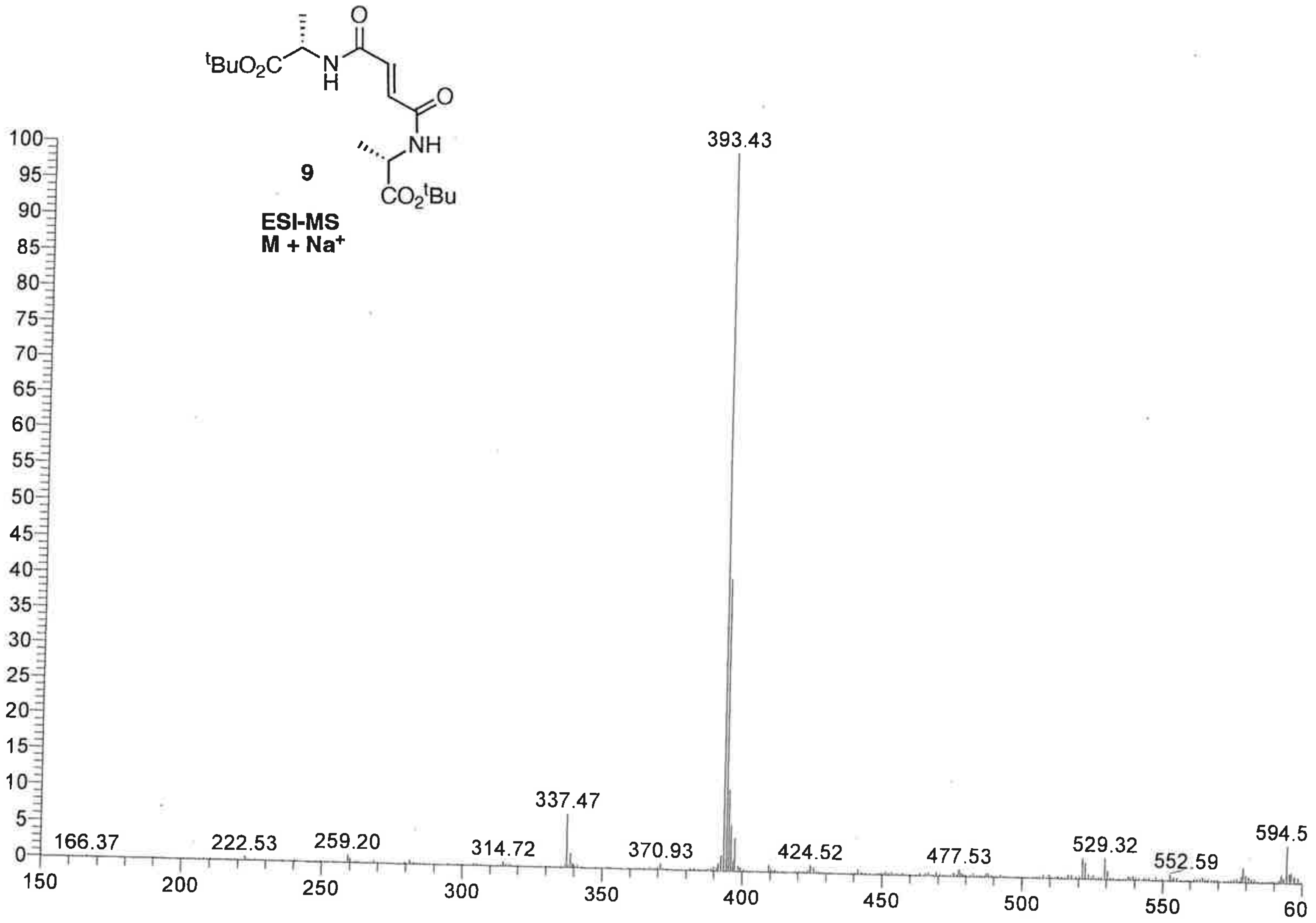

$^{t}BuO_2C$
N
H
O
O
NH
$CO_2{}^{t}Bu$
9
ESI-MS
M + Na^{+}
393.43
337.47
166.37
222.53
259.20
314.72
370.93
424.52
477.53
529.32
552.59
594.5
100
95
90
85
80
75
70
65
60
55
50
45
40
35
30
25
20
15
10
5
0
150
200
250
300
350
400
450
500
550
60

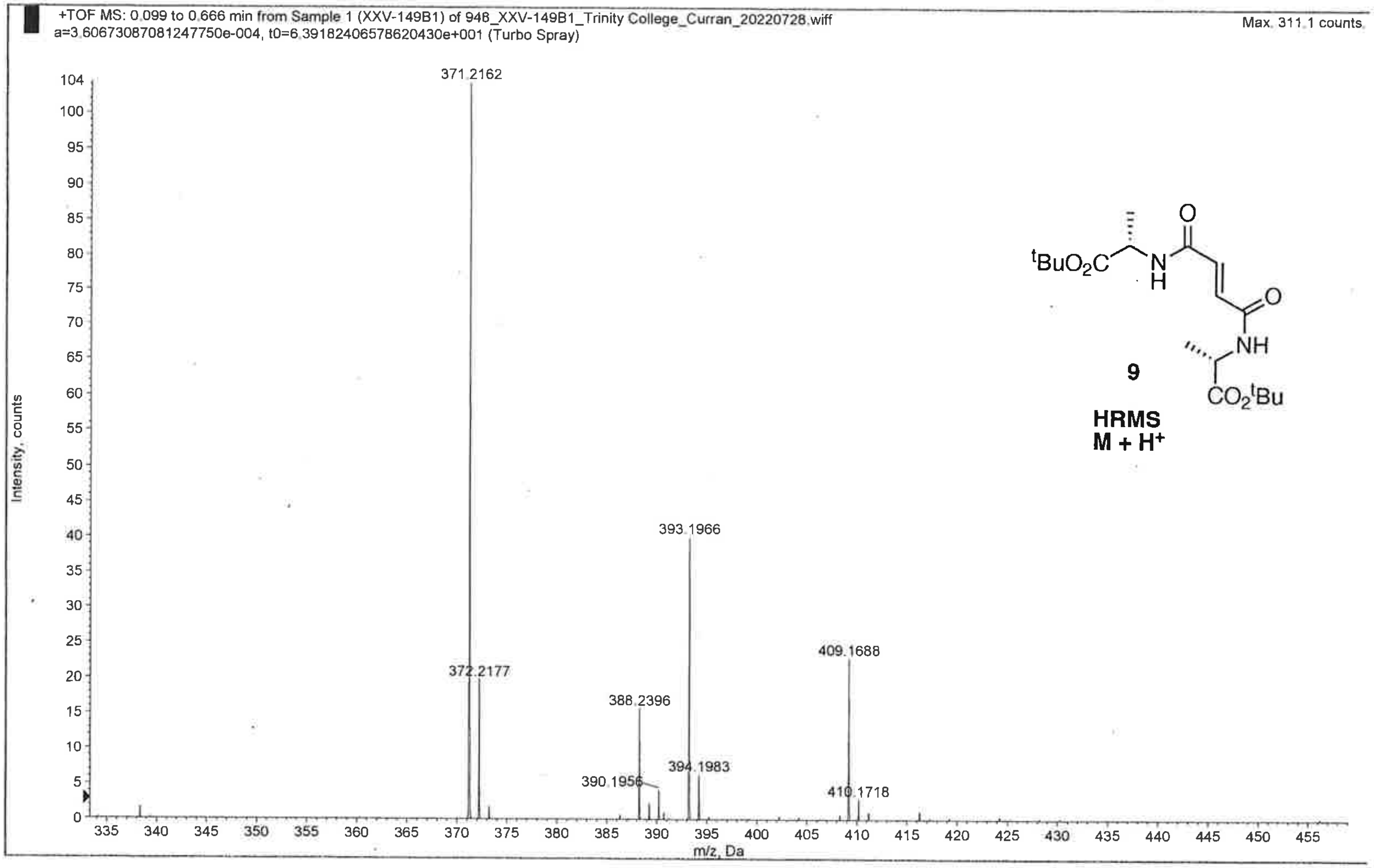

+TOF MS: 0.099 to 0.666 min from Sample 1 (XXV-149B1) of 948_XXV-149B1_Trinity College_Curran_20220728.wiff
a=3.60673087081247750e-004, t0=6.39182406578620430e+001 (Turbo Spray)
Max. 311.1 counts.
371.2162
372.2177
388.2396
390.1956
393.1966
394.1983
409.1688
410.1718
Intensity, counts
m/z, Da
$^{t}BuO_2C$
N
H
O
O
NH
$CO_2{}^{t}Bu$

9

HRMS
M + H$^+$

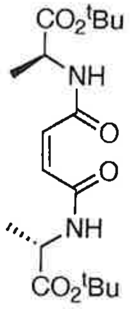

10

^{1}H NMR ($CDCl_3$)
100 MHz

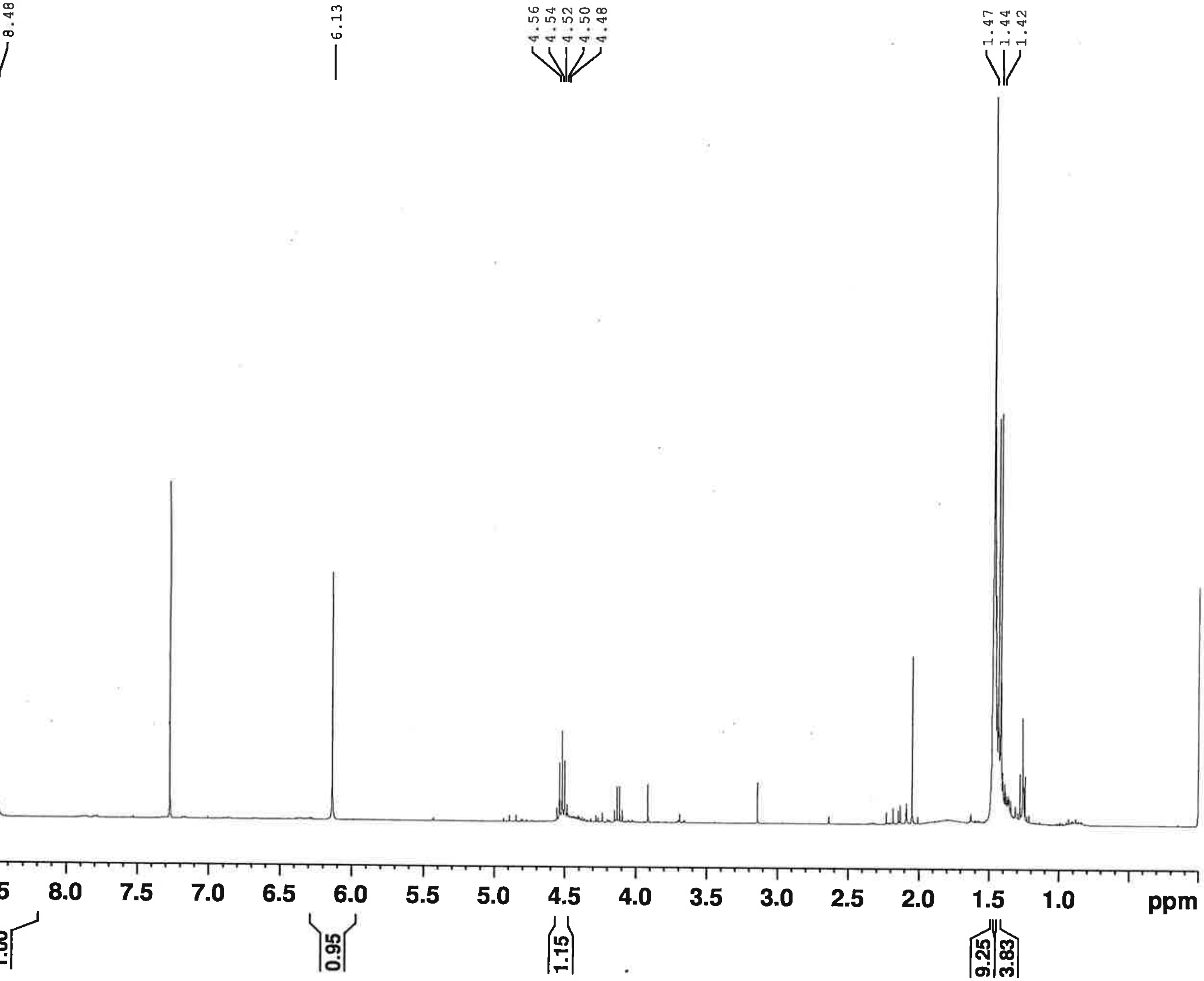

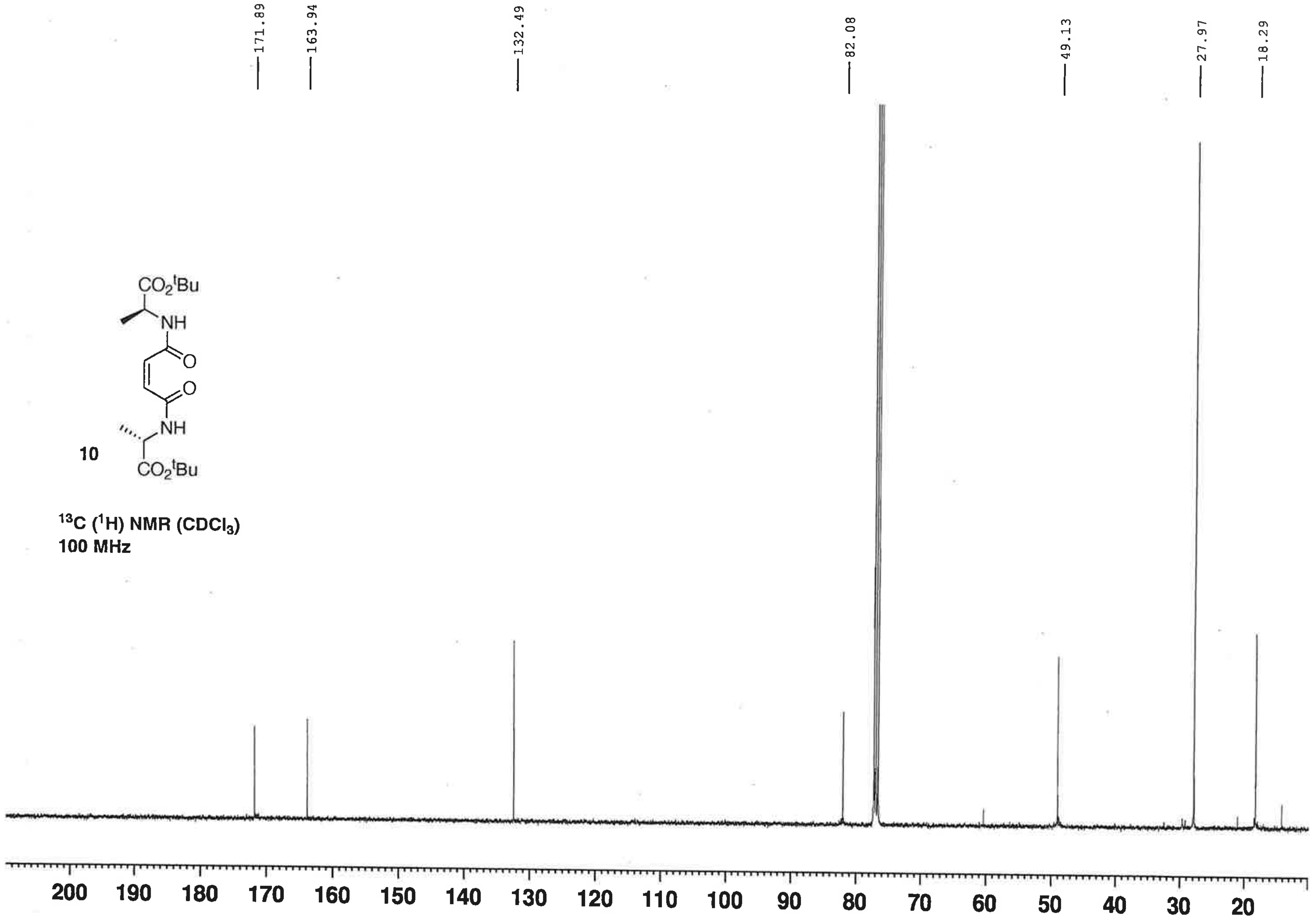

10

^{13}C (^1H) NMR ($CDCl_3$)
100 MHz

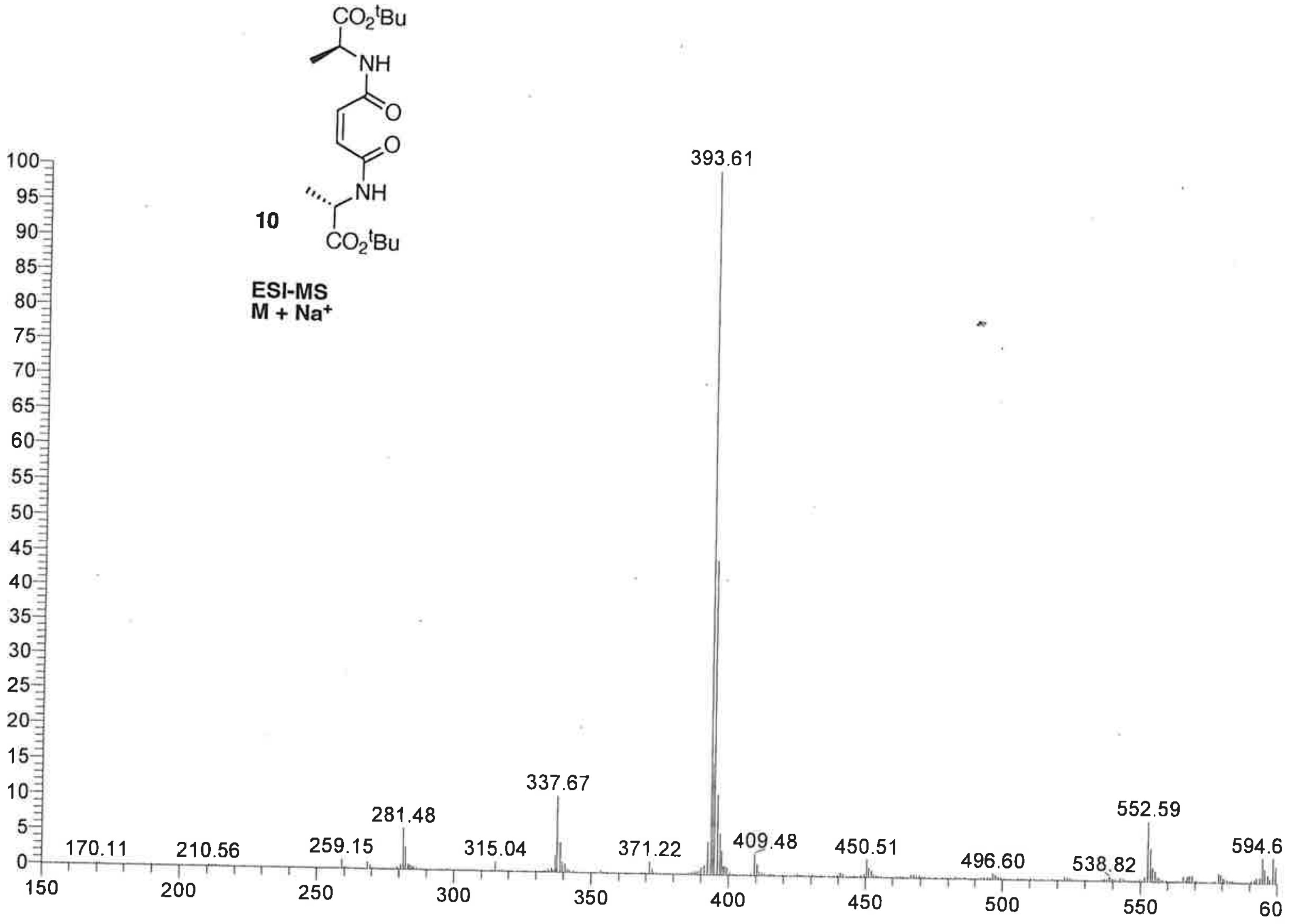

$CO_2{}^tBu$
NH
O
O
NH
10
$CO_2{}^tBu$
ESI-MS
M + Na+
393.61
337.67
281.48
170.11
210.56
259.15
315.04
371.22
409.48
450.51
496.60
538.82
552.59
594.6
100
95
90
85
80
75
70
65
60
55
50
45
40
35
30
25
20
15
10
5
0
150
200
250
300
350
400
450
500
550
60

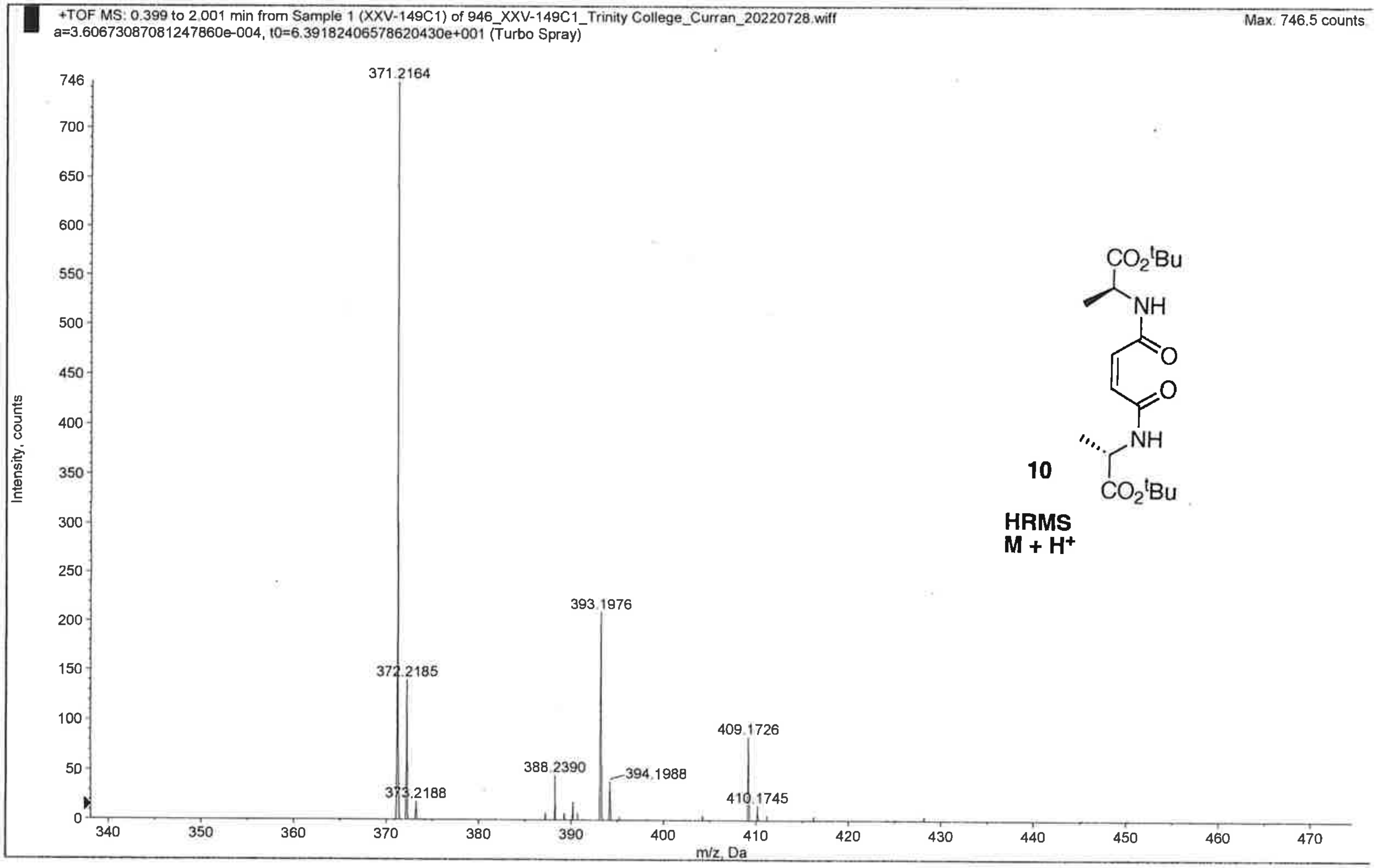
+TOF MS: 0.399 to 2.001 min from Sample 1 (XXV-149C1) of 946_XXV-149C1_Trinity College_Curran_20220728.wiff
a=3.60673087081247860e-004, t0=6.39182406578620430e+001 (Turbo Spray)
Max. 746.5 counts
Intensity, counts
m/z, Da
371.2164
372.2185
373.2188
388.2390
393.1976
394.1988
409.1726
410.1745
$CO_2{}^tBu$
NH
O
10
HRMS
M + H+

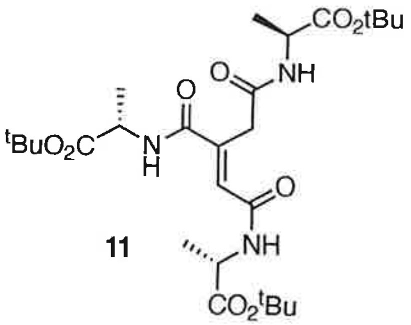

11

^{1}H NMR ($CDCl_3$)
100 MHz

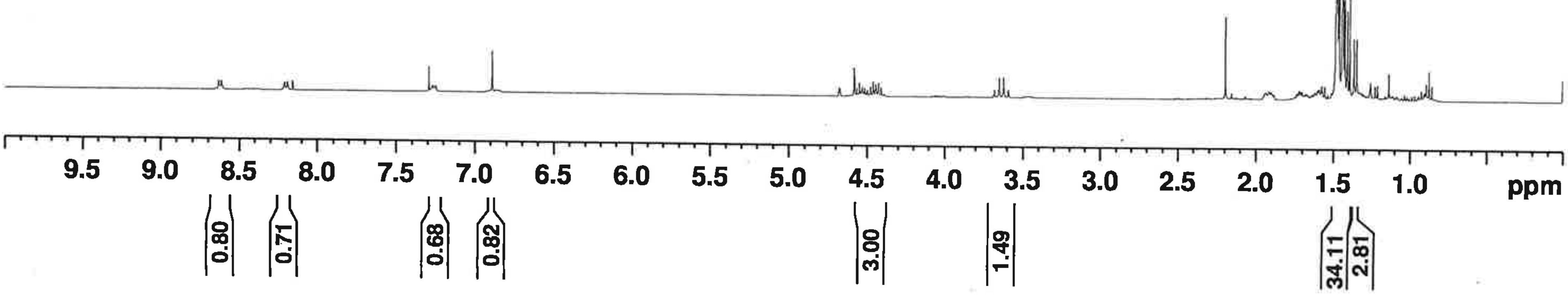

S21

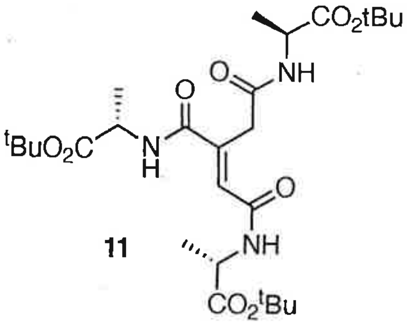

11

^{13}C (^1H) NMR ($CDCl_3$)
100 MHz

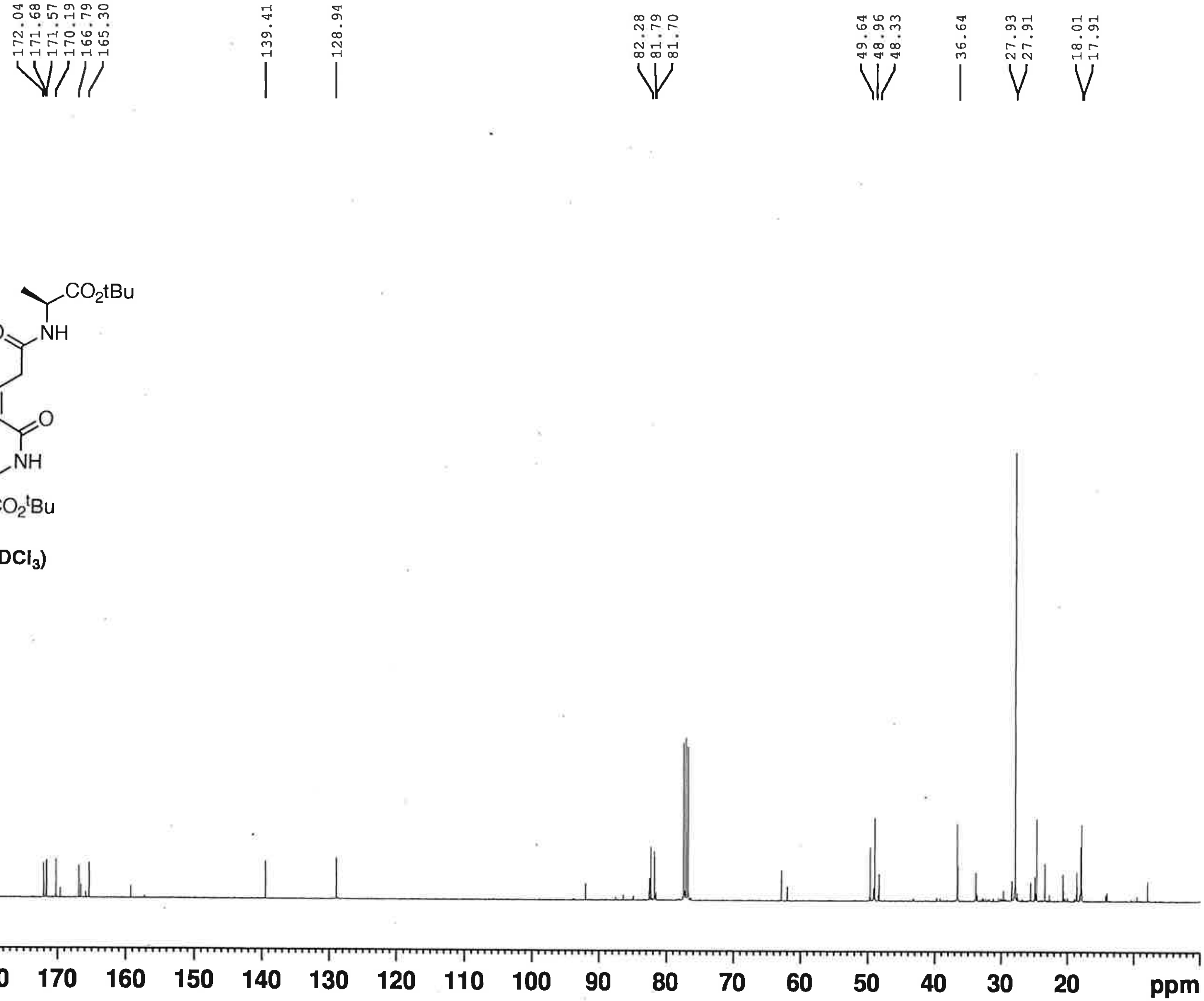

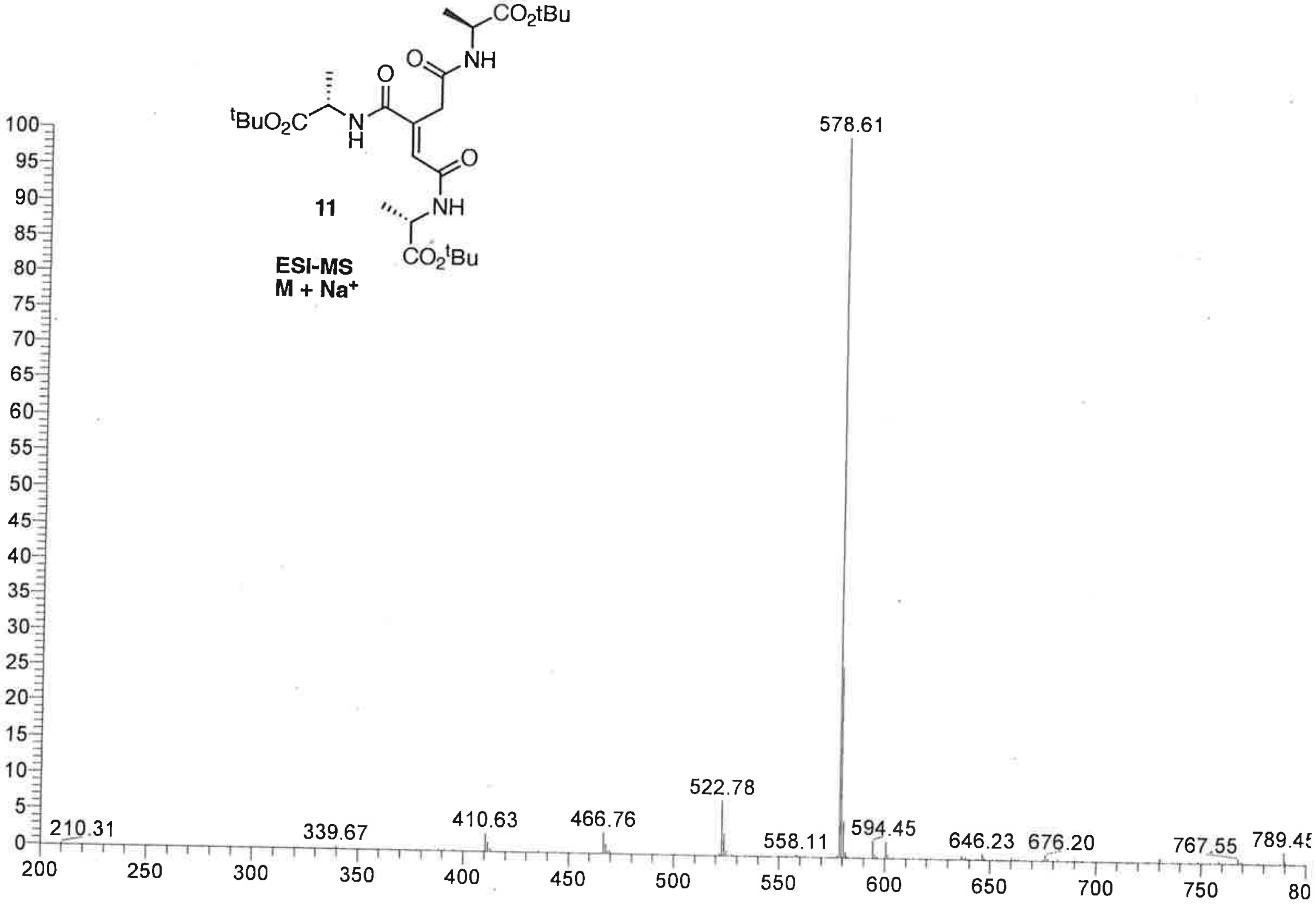

11
ESI-MS
M + Na+
578.61
522.78
466.76
410.63
339.67
210.31
558.11
594.45
646.23
676.20
767.55
789.45

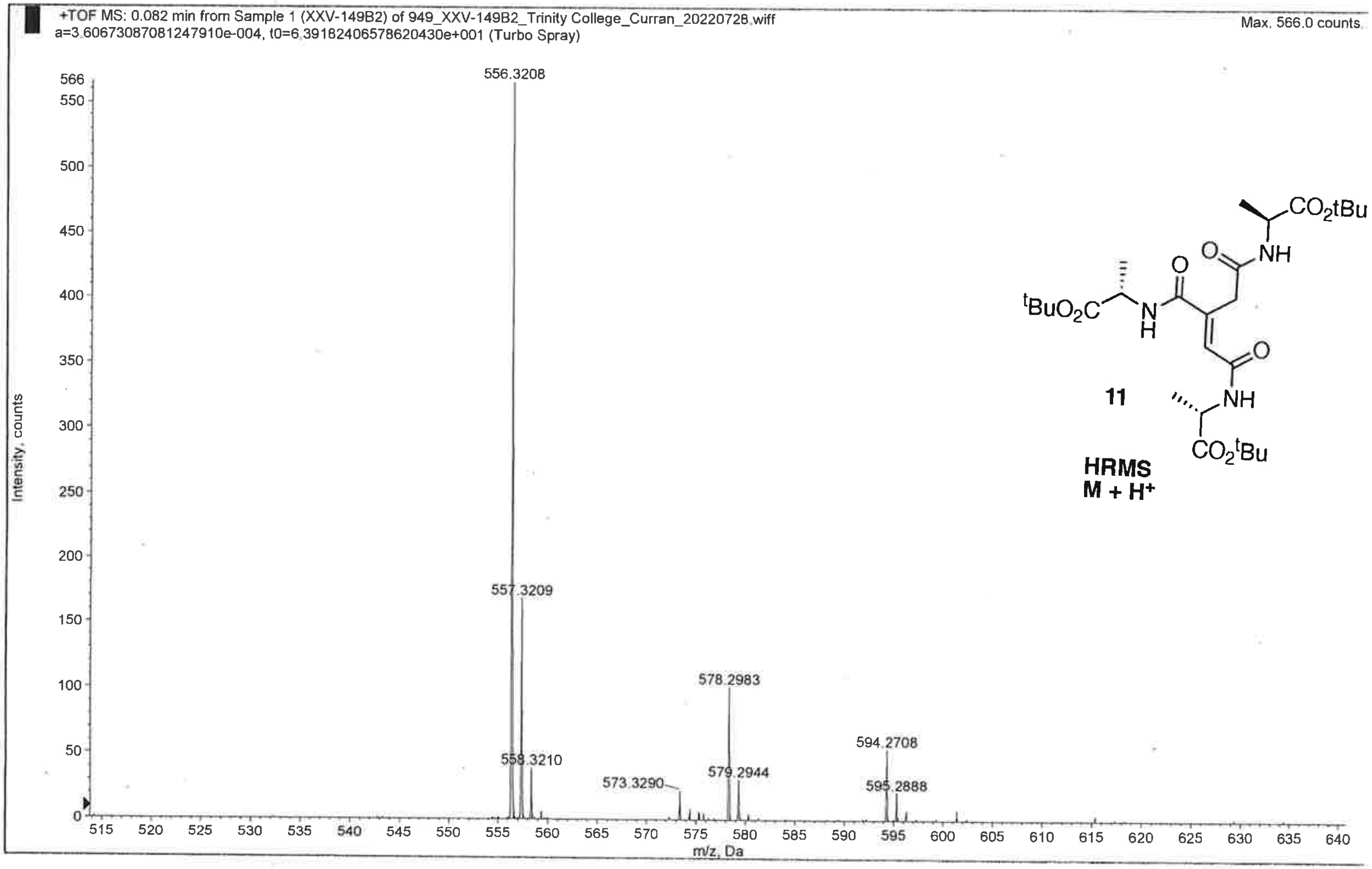
+TOF MS: 0.082 min from Sample 1 (XXV-149B2) of 949_XXV-149B2_Trinity College_Curran_20220728.wiff
a=3.60673087081247910e-004, t0=6.39182406578620430e+001 (Turbo Spray)
Max. 566.0 counts.
Intensity, counts
m/z, Da
556.3208
557.3209
558.3210
573.3290
578.2983
579.2944
594.2708
595.2888
CO2tBu
NH
tBuO2C
N H
NH
CO2tBu
11
HRMS
M + H+

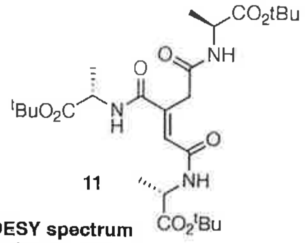

11

NOESY spectrum
$CDCl_3$: 400 MHz

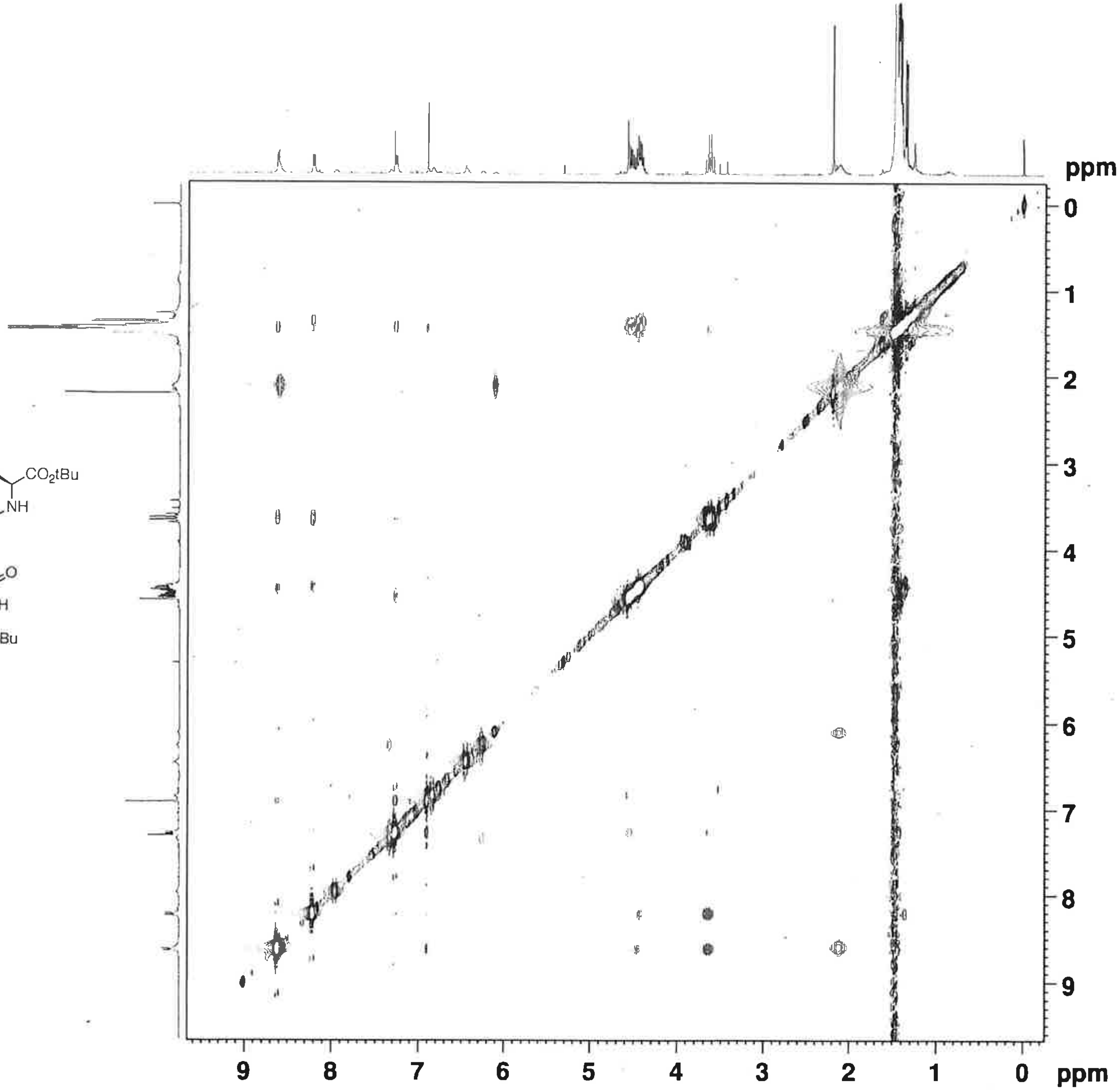

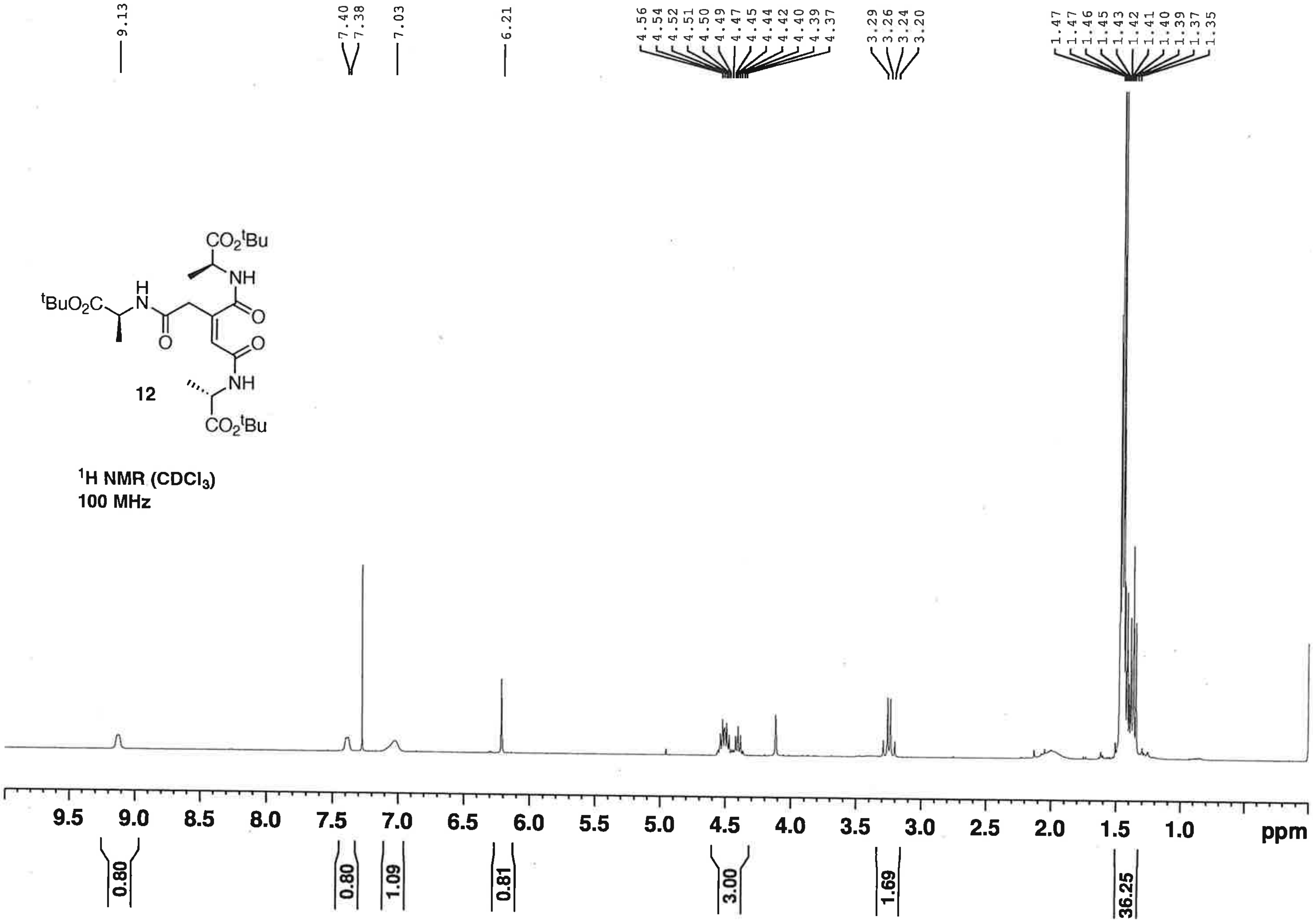

12

^{1}H NMR ($CDCl_3$)
100 MHz

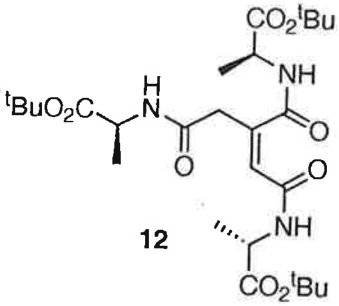

12

^{13}C (^{1}H) NMR ($CDCl_3$)
100 MHz

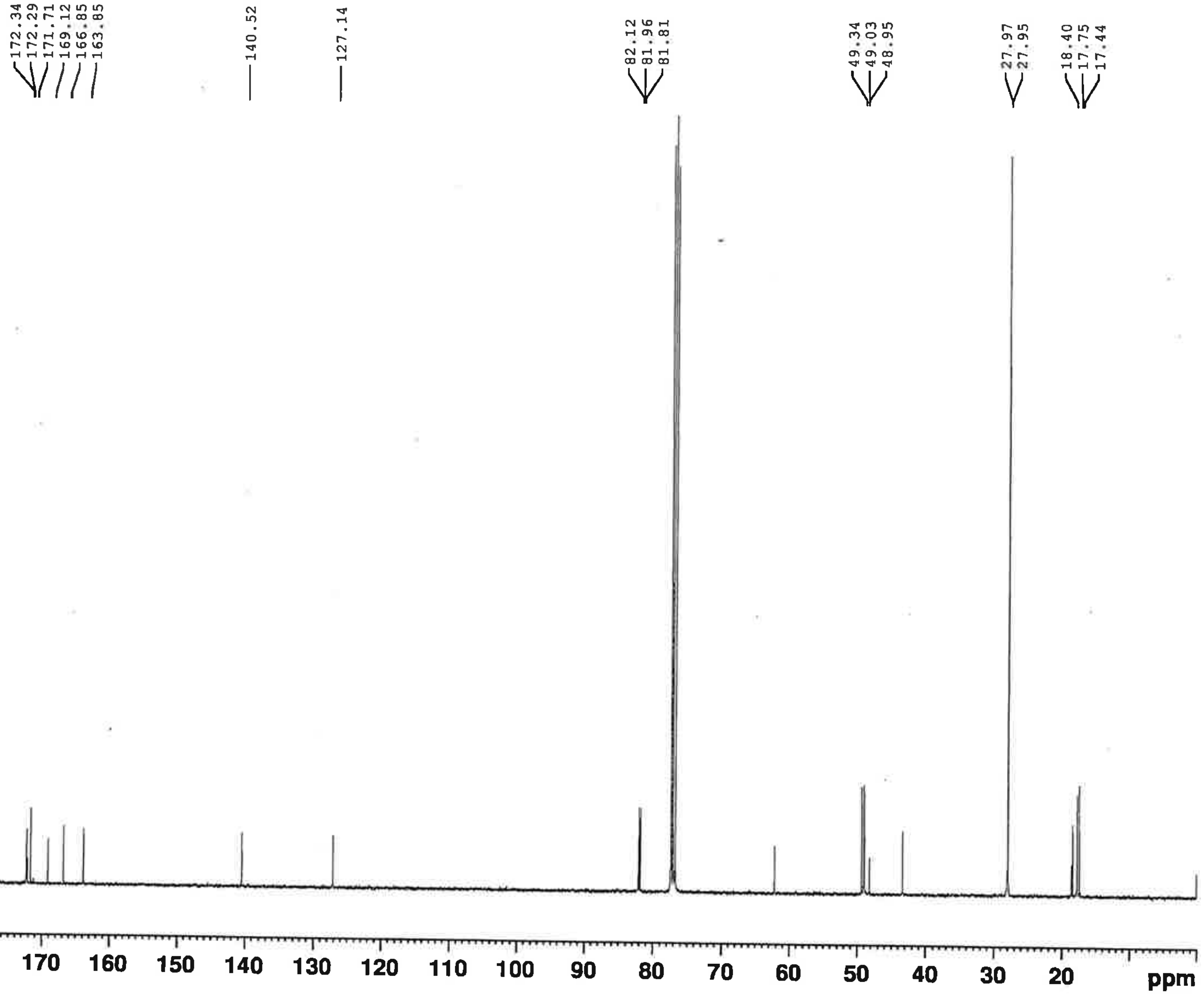

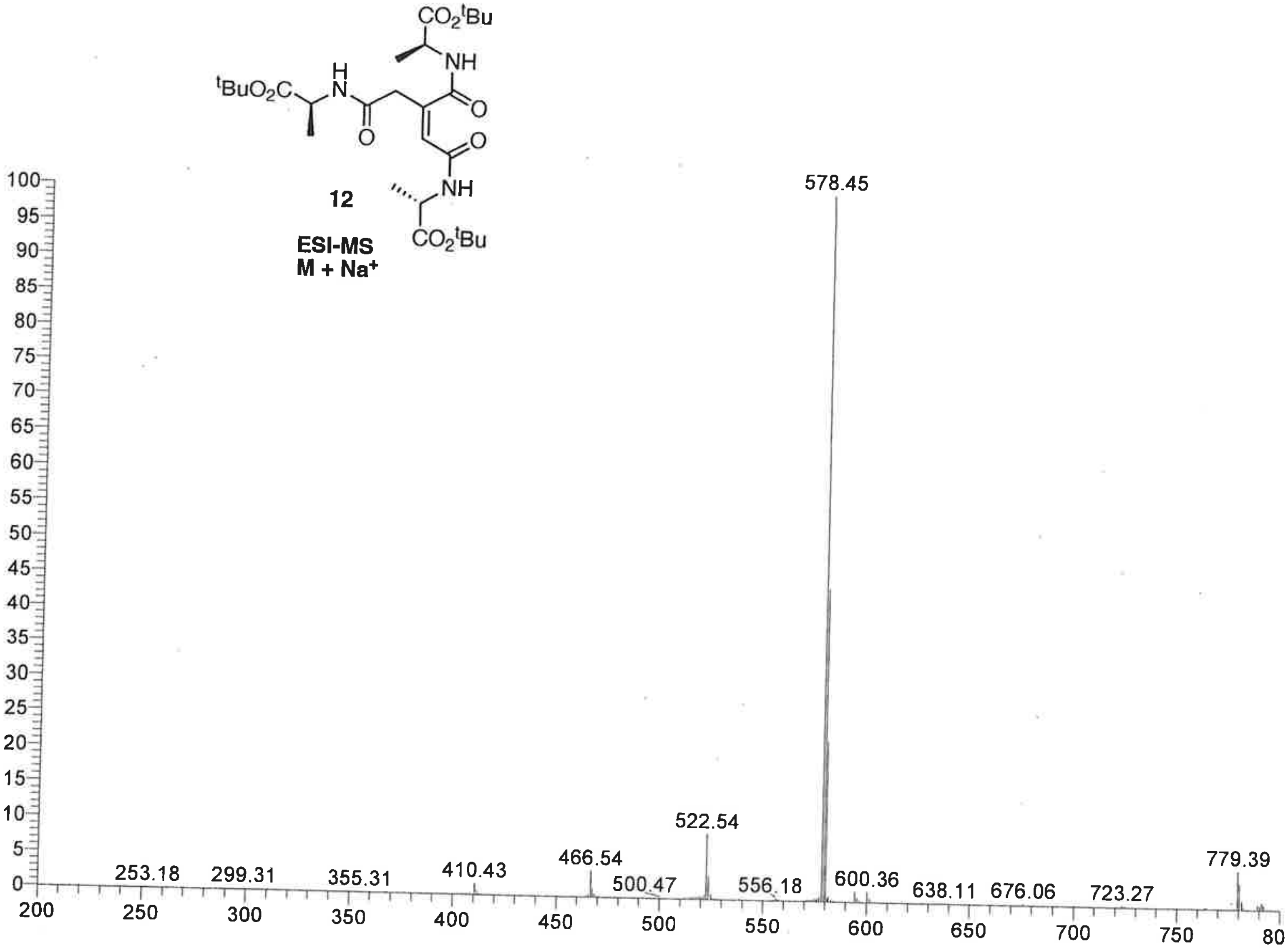
$CO_2{}^tBu$
NH
$^tBuO_2C$
H
N
O
O
O
NH
$CO_2{}^tBu$
12
ESI-MS
M + Na+
578.45
522.54
466.54
410.43
355.31
299.31
253.18
500.47
556.18
600.36
638.11
676.06
723.27
779.39
100
95
90
85
80
75
70
65
60
55
50
45
40
35
30
25
20
15
10
5
0
200
250
300
350
400
450
500
550
600
650
700
750
80

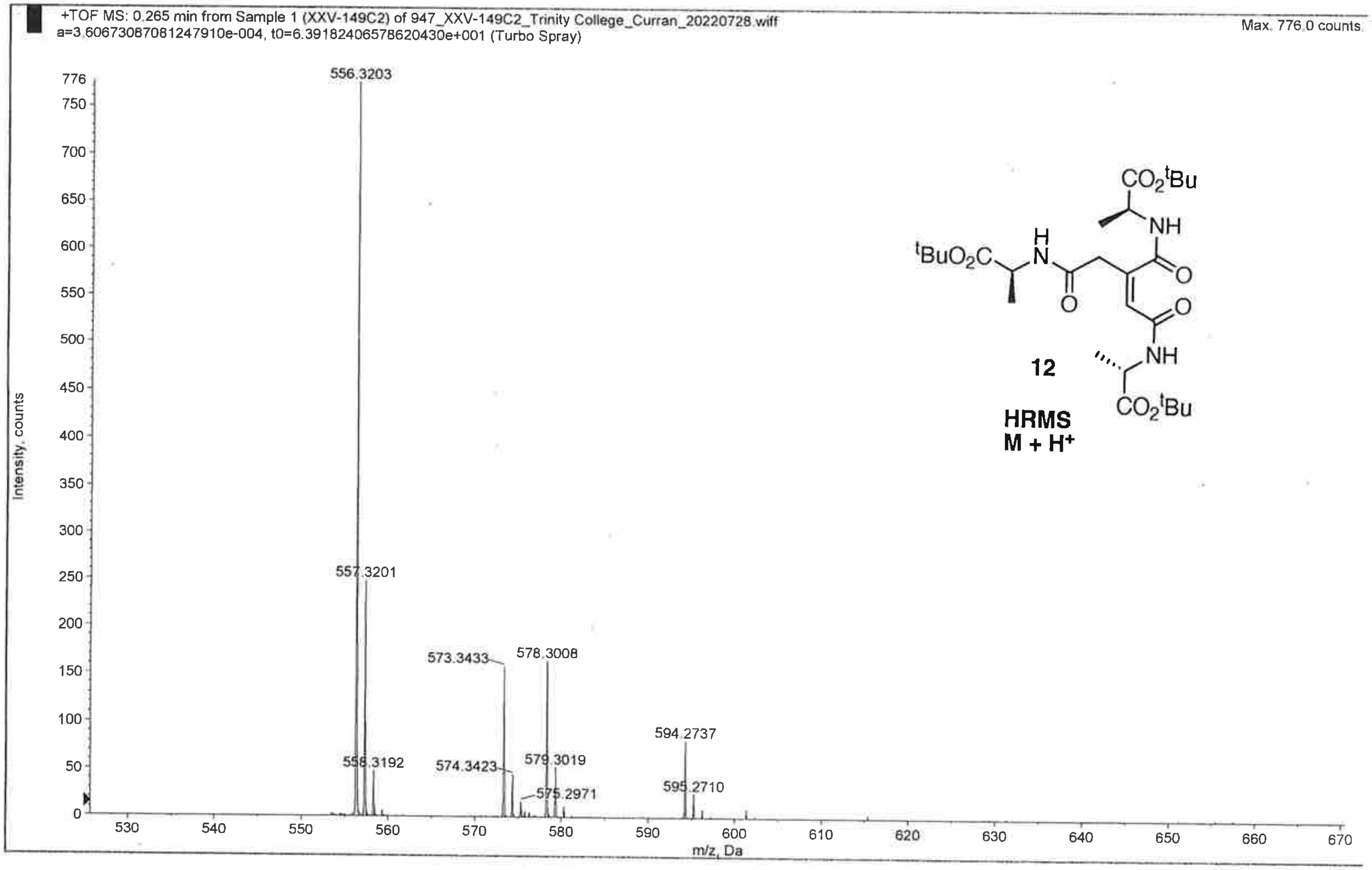
+TOF MS: 0.265 min from Sample 1 (XXV-149C2) of 947_XXV-149C2_Trinity College_Curran_20220728.wiff
a=3.60673087081247910e-004, t0=6.39182406578620430e+001 (Turbo Spray)
Max. 776.0 counts.
Intensity, counts
m/z, Da
556.3203
557.3201
558.3192
573.3433
574.3423
575.2971
578.3008
579.3019
594.2737
595.2710
CO2tBu
NH
tBuO2C
H
N
O
O
O
NH
CO2tBu

12

HRMS
M + H+

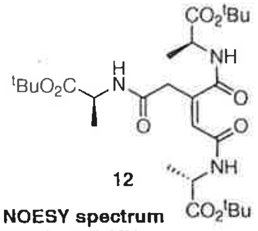

12

NOESY spectrum
$CDCl_3$: 400 MHz

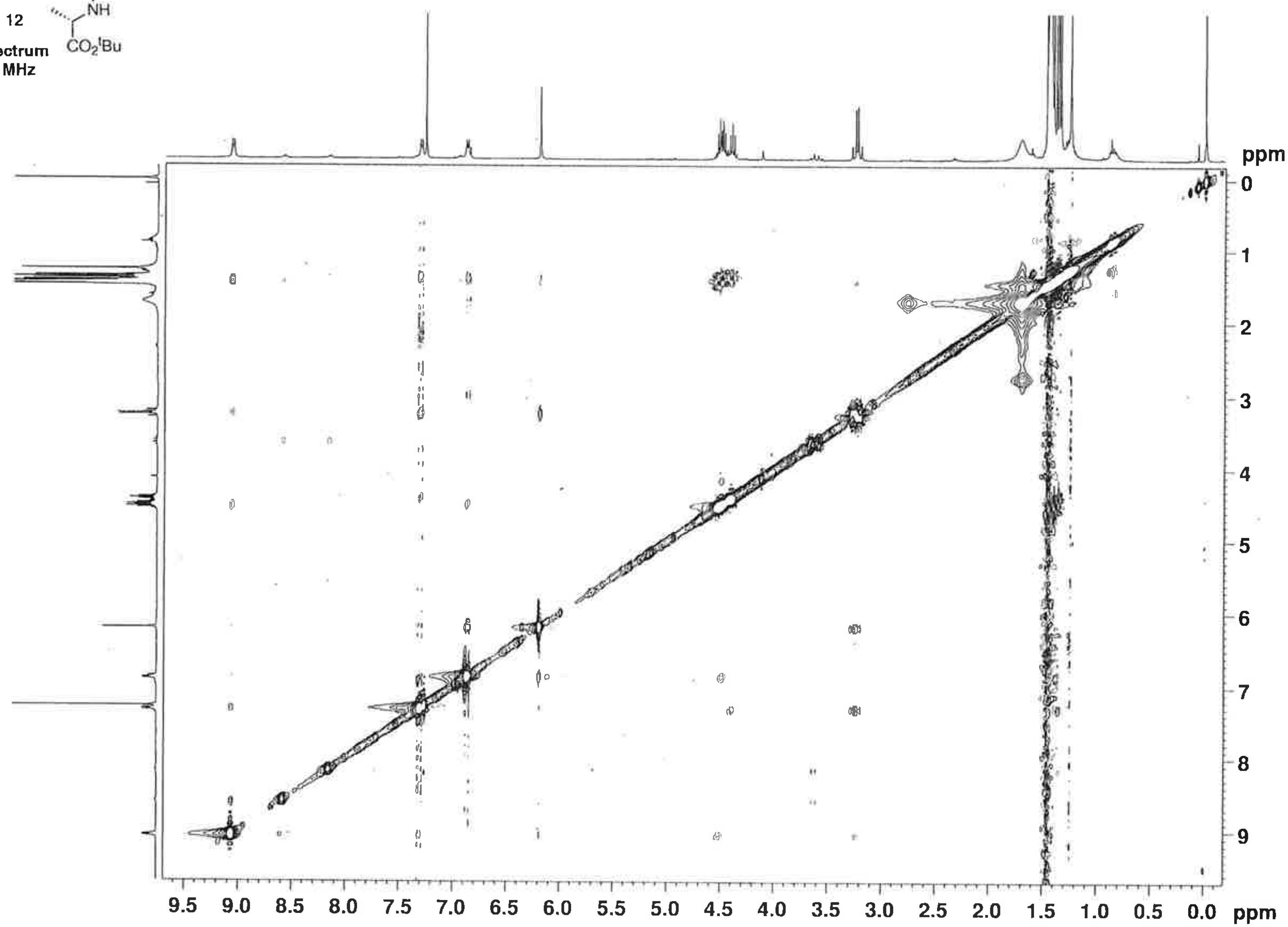

Note S1 - Z-matrices of the DFT optimized representative (three) conformations of **6**

```
#6.1
 c
 c   1 cc2
 o    2 oc3         1 occ3
 c    3 co4         2 coc4          1 dih4
 o    4 oc5         3 oco5          2 dih5
 c    4 cc6         3 cco6          2 dih6
 n    6 nc7         4 ncc7          3 dih7
 c    7 cn8         6 cnc8          4 dih8
 o    8 oc9         7 ocn9          6 dih9
 c    8 cc10        7 ccn10         6 dih10
 c   10 cc11        8 ccc11         7 dih11
 c   11 cc12       10 ccc12         8 dih12
 n   12 nc13       11 ncc13        10 dih13
 c   13 cn14       12 cnc14        11 dih14
 c   14 cc15       13 ccn15        12 dih15
 o   15 oc16       14 occ16        13 dih16
 c   10 cc17        8 ccc17         9 dih17
 c   17 cc18       10 ccc18         8 dih18
 n   18 nc19       17 ncc19        10 dih19
 c   19 cn20       18 cnc20        17 dih20
 c   20 cc21       19 ccn21        18 dih21
 o   21 oc22       20 occ22        19 dih22
 o   18 oc23       17 occ23        10 dih23
 o   12 oc24       11 occ24        10 dih24
 o   21 oc25       20 occ25        19 dih25
 c   25 co26       21 coc26        20 dih26
 c   26 cc27       25 cco27        21 dih27
 o   15 oc28       14 occ28        13 dih28
 c   28 co29       15 coc29        14 dih29
 c   29 cc30       28 cco30        15 dih30
 h   17 hc31       10 hcc31         8 dih31
 h   11 hc32       10 hcc32        17 dih32
 h   11 hc33       10 hcc33        17 dih33
 h   13 hn34       12 hnc34        11 dih34
 h    7 hn35        8 hnc35        10 dih35
 h   19 hn36       18 hnc36        17 dih36
 h   20 hc37       19 hcn37        18 dih37
 h   20 hc38       19 hcn38        18 dih38
 h   14 hc39       13 hcn39        12 dih39
 h   14 hc40       13 hcn40        12 dih40
 h    6 hc41        7 hcn41         8 dih41
 h    6 hc42        7 hcn42         8 dih42
 h    2 hc43        3 hco43         4 dih43
 h    2 hc44        3 hco44         4 dih44
 h    1 hc45        2 hcc45         3 dih45
 h    1 hc46        2 hcc46         3 dih46
 h    1 hc47        2 hcc47         3 dih47
 h   26 hc48       25 hco48        21 dih48
 h   26 hc49       25 hco49        21 dih49
 h   27 hc50       26 hcc50        25 dih50
 h   27 hc51       26 hcc51        25 dih51
 h   27 hc52       26 hcc52        25 dih52
 h   29 hc53       28 hco53        15 dih53
 h   29 hc54       28 hco54        15 dih54
 h   30 hc55       29 hcc55        28 dih55
 h   30 hc56       29 hcc56        28 dih56
 h   30 hc57       29 hcc57        28 dih57

cc2=        1.514509
oc3=        1.446179
occ3=       111.253
co4=        1.328949
coc4=       117.895
dih4=       -83.547
oc5=        1.204906
oco5=       124.892
dih5=        -1.887
cc6=        1.521380
```

```
cco6=       109.893
dih6=       177.596
nc7=        1.435093
ncc7=       112.897
dih7=       172.122
cn8=        1.347317
cnc8=       120.843
dih8=       -78.731
oc9=        1.226354
ocn9=       122.944
dih9=        -0.930
cc10=       1.513819
ccn10=      115.285
dih10=     -179.368
cc11=       1.513877
ccc11=      118.488
dih11=      -33.320
cc12=       1.523357
ccc12=      109.939
dih12=       88.913
nc13=       1.344700
ncc13=      115.487
dih13=       97.899
cn14=       1.441988
cnc14=      119.715
dih14=     -163.224
cc15=       1.524813
ccn15=      113.609
dih15=       68.053
oc16=       1.207880
occ16=      123.017
dih16=     -162.790
cc17=       1.335450
ccc17=      115.655
dih17=      -32.645
cc18=       1.484109
ccc18=      126.661
dih18=     -176.627
nc19=       1.351512
ncc19=      114.286
dih19=     -175.660
cn20=       1.438740
cnc20=      120.398
dih20=     -173.105
cc21=       1.523448
ccn21=      111.755
dih21=       71.576
oc22=       1.205618
occ22=      124.261
dih22=       26.566
oc23=       1.231219
occ23=      124.870
dih23=        4.766
oc24=       1.229785
occ24=      121.340
dih24=      -79.963
oc25=       1.327679
occ25=      110.803
dih25=     -154.848
co26=       1.447062
coc26=      117.413
dih26=      179.926
cc27=       1.514561
cco27=      111.111
dih27=      -80.904
oc28=       1.323617
occ28=      111.582
dih28=       18.607
co29=       1.442766
coc29=      119.168
dih29=     -176.960
```

```
cc30=       1.514306
cco30=      110.005
dih30=       91.037
hc31=       1.085883
hcc31=      116.961
dih31=        2.474
hc32=       1.090747
hcc32=      109.731
dih32=      151.415
hc33=       1.087212
hcc33=      109.712
dih33=       31.711
hn34=       1.016717
hnc34=      115.124
dih34=      -13.458
hn35=       1.018272
hnc35=      119.718
dih35=       -3.849
hn36=       1.007661
hnc36=      119.657
dih36=       -4.897
hc37=       1.090935
hcn37=      109.116
dih37=     -168.971
hc38=       1.089765
hcn38=      110.475
dih38=      -50.257
hc39=       1.090951
hcn39=      109.148
dih39=     -171.653
hc40=       1.089279
hcn40=      110.724
dih40=      -53.103
hc41=       1.091698
hcn41=      110.954
dih41=       42.963
hc42=       1.091715
hcn42=      109.177
dih42=      161.318
hc43=       1.091431
hco43=      108.641
dih43=       39.878
hc44=       1.088632
hco44=      104.157
dih44=      156.129
hc45=       1.092617
hcc45=      109.164
dih45=     -176.529
hc46=       1.091537
hcc46=      110.709
dih46=      -57.313
hc47=       1.090514
hcc47=      111.103
dih47=       64.078
hc48=       1.089953
hco48=      108.769
dih48=       42.537
hc49=       1.088887
hco49=      104.078
dih49=      158.885
hc50=       1.092047
hcc50=      110.727
dih50=      -56.880
hc51=       1.091209
hcc51=      111.029
dih51=       64.467
hc52=       1.092851
hcc52=      109.222
dih52=     -176.134
hc53=       1.089011
hco53=      104.124
```

```
dih53=     -148.599
hc54=       1.090511
hco54=      109.064
dih54=      -32.052
hc55=       1.092956
hcc55=      111.026
dih55=      -63.587
hc56=       1.093035
hcc56=      110.653
dih56=       57.187
hc57=       1.092121
hcc57=      109.576
dih57=      176.740

#6.2
 c
 c   1 cc2
 o    2 oc3         1 occ3
 c    3 co4         2 coc4           1 dih4
 o    4 oc5         3 oco5           2 dih5
 c    4 cc6         3 cco6           2 dih6
 n    6 nc7         4 ncc7           3 dih7
 c    7 cn8         6 cnc8           4 dih8
 o    8 oc9         7 ocn9           6 dih9
 c    8 cc10        7 ccn10          6 dih10
 c   10 cc11        8 ccc11          7 dih11
 c   11 cc12       10 ccc12          8 dih12
 n   12 nc13       11 ncc13         10 dih13
 c   13 cn14       12 cnc14         11 dih14
 c   14 cc15       13 ccn15         12 dih15
 o   15 oc16       14 occ16         13 dih16
 c   10 cc17        8 ccc17          9 dih17
 c   17 cc18       10 ccc18          8 dih18
 n   18 nc19       17 ncc19         10 dih19
 c   19 cn20       18 cnc20         17 dih20
 c   20 cc21       19 ccn21         18 dih21
 o   21 oc22       20 occ22         19 dih22
 o   18 oc23       17 occ23         10 dih23
 o   12 oc24       11 occ24         10 dih24
 o   21 oc25       20 occ25         19 dih25
 c   25 co26       21 coc26         20 dih26
 c   26 cc27       25 cco27         21 dih27
 o   15 oc28       14 occ28         13 dih28
 c   28 co29       15 coc29         14 dih29
 c   29 cc30       28 cco30         15 dih30
 h   17 hc31       10 hcc31          8 dih31
 h   11 hc32       10 hcc32         17 dih32
 h   11 hc33       10 hcc33         17 dih33
 h   13 hn34       12 hnc34         11 dih34
 h    7 hn35        8 hnc35         10 dih35
 h   19 hn36       18 hnc36         17 dih36
 h   20 hc37       19 hcn37         18 dih37
 h   20 hc38       19 hcn38         18 dih38
 h   14 hc39       13 hcn39         12 dih39
 h   14 hc40       13 hcn40         12 dih40
 h    6 hc41        7 hcn41          8 dih41
 h    6 hc42        7 hcn42          8 dih42
 h    2 hc43        3 hco43          4 dih43
 h    2 hc44        3 hco44          4 dih44
 h    1 hc45        2 hcc45          3 dih45
 h    1 hc46        2 hcc46          3 dih46
 h    1 hc47        2 hcc47          3 dih47
 h   26 hc48       25 hco48         21 dih48
 h   26 hc49       25 hco49         21 dih49
 h   27 hc50       26 hcc50         25 dih50
 h   27 hc51       26 hcc51         25 dih51
 h   27 hc52       26 hcc52         25 dih52
 h   29 hc53       28 hco53         15 dih53
 h   29 hc54       28 hco54         15 dih54
 h   30 hc55       29 hcc55         28 dih55
 h   30 hc56       29 hcc56         28 dih56
```

```
 h   30 hc57       29 hcc57        28 dih57

cc2         1.510116
oc3         1.444853
occ3        107.475
co4         1.321456
coc4        117.552
dih4       -170.246
oc5         1.208743
oco5        124.740
dih5         -1.244
cc6         1.523376
cco6        113.244
dih6        179.518
nc7         1.438262
ncc7        115.471
dih7         -6.097
cn8         1.351123
cnc8        120.338
dih8        -73.006
oc9         1.224648
ocn9        122.825
dih9         -7.601
cc10        1.513343
ccn10       115.277
dih10       171.563
cc11        1.511408
ccc11       118.780
dih11        26.413
cc12        1.525152
ccc12       110.512
dih12       -86.558
nc13        1.342838
ncc13       115.293
dih13       -94.064
cn14        1.436982
cnc14       121.098
dih14       170.302
cc15        1.522511
ccn15       112.453
dih15       -74.053
oc16        1.204855
occ16       124.915
dih16        -6.327
cc17        1.336862
ccc17       115.644
dih17        27.295
cc18        1.484722
ccc18       126.464
dih18       175.657
nc19        1.349225
ncc19       114.442
dih19       176.773
cn20        1.439996
cnc20       120.400
dih20       173.131
cc21        1.522874
ccn21       114.623
dih21       -63.689
oc22        1.208273
occ22       122.228
dih22       158.685
oc23        1.232611
occ23       124.825
dih23        -4.341
oc24        1.230557
occ24       121.343
dih24        84.892
oc25        1.324103
occ25       112.699
dih25       -22.792
```

co26	1.445740
coc26	118.055
dih26	179.630
cc27	1.512970
cco27	111.325
dih27	84.813
oc28	1.326849
occ28	110.545
dih28	174.498
co29	1.446172
coc29	116.860
dih29	179.442
cc30	1.509629
cco30	107.638
dih30	-179.747
hc31	1.085574
hcc31	116.958
dih31	-3.023
hc32	1.087826
hcc32	109.677
dih32	-30.162
hc33	1.090321
hcc33	109.929
dih33	-149.893
hn34	1.016040
hnc34	116.419
dih34	10.219
hn35	1.018568
hnc35	118.975
dih35	11.147
hn36	1.007495
hnc36	119.621
dih36	4.708
hc37	1.090773
hcn37	110.798
dih37	57.461
hc38	1.091114
hcn38	108.705
dih38	175.785
hc39	1.091460
hcn39	110.454
dih39	47.325
hc40	1.090746
hcn40	109.436
dih40	165.559
hc41	1.090403
hcn41	110.383
dih41	48.371
hc42	1.091188
hcn42	109.087
dih42	166.303
hc43	1.091238
hco43	108.513
dih43	-48.948
hc44	1.091921
hco44	108.132
dih44	68.567
hc45	1.092044
hcc45	109.161
dih45	-178.200
hc46	1.090405
hcc46	111.285
dih46	-58.127
hc47	1.091632
hcc47	110.726
dih47	62.568
hc48	1.088599
hco48	103.706
dih48	-154.956
hc49	1.091268
hco49	108.617

```
dih49       -38.755
hc50        1.092678
hcc50       109.182
dih50       176.318
hc51        1.091249
hcc51       111.200
dih51       -64.273
hc52        1.091556
hcc52       110.756
dih52        57.141
hc53        1.092525
hco53       108.505
dih53       -58.502
hc54        1.091628
hco54       108.400
dih54        58.991
hc55        1.091068
hcc55       109.218
dih55      -179.929
hc56        1.092251
hcc56       110.915
dih56       -60.581
hc57        1.092030
hcc57       110.979
dih57        60.610

#6.3
 c
 c   1 cc2
 o    2 oc3         1 occ3
 c    3 co4         2 coc4          1 dih4
 o    4 oc5         3 oco5          2 dih5
 c    4 cc6         3 cco6          2 dih6
 n    6 nc7         4 ncc7          3 dih7
 c    7 cn8         6 cnc8          4 dih8
 o    8 oc9         7 ocn9          6 dih9
 c    8 cc10        7 ccn10         6 dih10
 c   10 cc11        8 ccc11         7 dih11
 c   11 cc12       10 ccc12         8 dih12
 n   12 nc13       11 ncc13        10 dih13
 c   13 cn14       12 cnc14        11 dih14
 c   14 cc15       13 ccn15        12 dih15
 o   15 oc16       14 occ16        13 dih16
 c   10 cc17        8 ccc17         9 dih17
 c   17 cc18       10 ccc18         8 dih18
 n   18 nc19       17 ncc19        10 dih19
 c   19 cn20       18 cnc20        17 dih20
 c   20 cc21       19 ccn21        18 dih21
 o   21 oc22       20 occ22        19 dih22
 o   18 oc23       17 occ23        10 dih23
 o   12 oc24       11 occ24        10 dih24
 o   21 oc25       20 occ25        19 dih25
 c   25 co26       21 coc26        20 dih26
 c   26 cc27       25 cco27        21 dih27
 o   15 oc28       14 occ28        13 dih28
 c   28 co29       15 coc29        14 dih29
 c   29 cc30       28 cco30        15 dih30
 h   17 hc31       10 hcc31         8 dih31
 h   11 hc32       10 hcc32        17 dih32
 h   11 hc33       10 hcc33        17 dih33
 h   13 hn34       12 hnc34        11 dih34
 h    7 hn35        8 hnc35        10 dih35
 h   19 hn36       18 hnc36        17 dih36
 h   20 hc37       19 hcn37        18 dih37
 h   20 hc38       19 hcn38        18 dih38
 h   14 hc39       13 hcn39        12 dih39
 h   14 hc40       13 hcn40        12 dih40
 h    6 hc41        7 hcn41         8 dih41
 h    6 hc42        7 hcn42         8 dih42
 h    2 hc43        3 hco43         4 dih43
 h    2 hc44        3 hco44         4 dih44
```

```
 h    1 hc45        2 hcc45         3 dih45
 h    1 hc46        2 hcc46         3 dih46
 h    1 hc47        2 hcc47         3 dih47
 h   26 hc48       25 hco48        21 dih48
 h   26 hc49       25 hco49        21 dih49
 h   27 hc50       26 hcc50        25 dih50
 h   27 hc51       26 hcc51        25 dih51
 h   27 hc52       26 hcc52        25 dih52
 h   29 hc53       28 hco53        15 dih53
 h   29 hc54       28 hco54        15 dih54
 h   30 hc55       29 hcc55        28 dih55
 h   30 hc56       29 hcc56        28 dih56
 h   30 hc57       29 hcc57        28 dih57

cc2         1.514005
oc3         1.445218
occ3        111.281
co4         1.325627
coc4        117.562
dih4         80.730
oc5         1.208951
oco5        124.741
dih5          3.386
cc6         1.521679
cco6        113.319
dih6       -178.370
nc7         1.438017
ncc7        115.758
dih7         15.887
cn8         1.348475
cnc8        120.196
dih8         67.464
oc9         1.227007
ocn9        122.683
dih9          4.014
cc10        1.512446
ccn10       115.403
dih10      -175.041
cc11        1.512451
ccc11       118.686
dih11       -29.224
cc12        1.523371
ccc12       110.140
dih12        87.718
nc13        1.342610
ncc13       115.440
dih13        95.403
cn14        1.440148
cnc14       120.493
dih14      -167.885
cc15        1.522041
ccn15       114.708
dih15        68.758
oc16        1.207693
occ16       122.763
dih16      -168.163
cc17        1.335343
ccc17       115.560
dih17       -29.998
cc18        1.485738
ccc18       126.765
dih18      -175.819
nc19        1.350436
ncc19       113.756
dih19      -178.736
cn20        1.438385
cnc20       121.824
dih20      -174.385
cc21        1.523057
ccn21       115.689
dih21        76.781
```

oc22	1.206761
occ22	121.654
dih22	177.565
oc23	1.231234
occ23	124.746
dih23	1.820
oc24	1.230894
occ24	121.425
dih24	-82.664
oc25	1.323484
occ25	113.141
dih25	-2.980
co26	1.443943
coc26	118.271
dih26	-179.623
cc27	1.513929
cco27	110.526
dih27	-93.640
oc28	1.324602
occ28	112.623
dih28	13.370
co29	1.441427
coc29	117.712
dih29	179.966
cc30	1.510068
cco30	107.011
dih30	-179.246
hc31	1.085843
hcc31	116.808
dih31	2.828
hc32	1.090558
hcc32	109.824
dih32	151.075
hc33	1.086967
hcc33	109.662
dih33	31.327
hn34	1.016571
hnc34	116.181
dih34	-10.918
hn35	1.018806
hnc35	119.311
dih35	-10.297
hn36	1.006980
hnc36	119.269
dih36	-3.571
hc37	1.089695
hcn37	109.235
dih37	-161.870
hc38	1.092011
hcn38	109.956
dih38	-44.089
hc39	1.091746
hcn39	109.043
dih39	-170.811
hc40	1.090016
hcn40	110.675
dih40	-52.645
hc41	1.091625
hcn41	108.752
dih41	-171.907
hc42	1.091370
hcn42	110.643
dih42	-53.983
hc43	1.089503
hco43	104.008
dih43	-159.105
hc44	1.090617
hco44	108.841
dih44	-42.920
hc45	1.091336
hcc45	111.091

```
dih45       -65.105
hc46         1.092091
hcc46       110.800
dih46        56.140
hc47         1.092188
hcc47       109.178
dih47       175.425
hc48         1.089695
hco48       108.997
dih48        29.721
hc49         1.089270
hco49       104.349
dih49       146.099
hc50         1.091673
hcc50       110.949
dih50        64.256
hc51         1.092620
hcc51       109.382
dih51      -176.198
hc52         1.092330
hcc52       110.839
dih52       -56.772
hc53         1.092843
hco53       108.789
dih53       -57.932
hc54         1.092223
hco54       108.434
dih54        59.435
hc55         1.091961
hcc55       110.510
dih55        58.686
hc56         1.092468
hcc56       109.522
dih56       178.403
hc57         1.091557
hcc57       110.526
dih57       -61.930
```

Note S2 - Z-matrices of the DFT optimized representative (four) conformations of **7**

```
#7.1
 c
 c   1 cc2
 o    2 oc3        1 occ3
 c    3 co4        2 coc4          1 dih4
 o    4 oc5        3 oco5          2 dih5
 c    4 cc6        3 cco6          2 dih6
 n    6 nc7        4 ncc7          3 dih7
 c    7 cn8        6 cnc8          4 dih8
 o    8 oc9        7 ocn9          6 dih9
 c    8 cc10       7 ccn10         6 dih10
 c   10 cc11       8 ccc11         7 dih11
 c   11 cc12      10 ccc12         8 dih12
 n   12 nc13      11 ncc13        10 dih13
 c   13 cn14      12 cnc14        11 dih14
 c   14 cc15      13 ccn15        12 dih15
 o   15 oc16      14 occ16        13 dih16
 c   11 cc17      10 ccc17         8 dih17
 c   17 cc18      11 ccc18        10 dih18
 n   18 nc19      17 ncc19        11 dih19
 c   19 cn20      18 cnc20        17 dih20
 c   20 cc21      19 ccn21        18 dih21
 o   21 oc22      20 occ22        19 dih22
 o   18 oc23      19 ocn23        20 dih23
 o   21 oc24      20 occ24        19 dih24
 c   24 co25      21 coc25        20 dih25
 c   25 cc26      24 cco26        21 dih26
 o   12 oc27      11 occ27        10 dih27
 o   15 oc28      14 occ28        13 dih28
 c   28 co29      15 coc29        14 dih29
 c   29 cc30      28 cco30        15 dih30
```

```
 h   10 hc31       11 hcc31        17 dih31
 h    7 hn32        8 hnc32        10 dih32
 h    6 hc33        7 hcn33         8 dih33
 h    6 hc34        7 hcn34         8 dih34
 h    2 hc35        3 hco35         4 dih35
 h    2 hc36        3 hco36         4 dih36
 h    1 hc37        2 hcc37         3 dih37
 h    1 hc38        2 hcc38         3 dih38
 h    1 hc39        2 hcc39         3 dih39
 h   19 hn40       18 hnc40        23 dih40
 h   20 hc41       19 hcn41        18 dih41
 h   20 hc42       19 hcn42        18 dih42
 h   25 hc43       24 hco43        21 dih43
 h   25 hc44       24 hco44        21 dih44
 h   26 hc45       25 hcc45        24 dih45
 h   26 hc46       25 hcc46        24 dih46
 h   26 hc47       25 hcc47        24 dih47
 h   17 hc48       11 hcc48        10 dih48
 h   17 hc49       11 hcc49        10 dih49
 h   13 hn50       12 hnc50        11 dih50
 h   14 hc51       13 hcn51        12 dih51
 h   14 hc52       13 hcn52        12 dih52
 h   29 hc53       28 hco53        15 dih53
 h   29 hc54       28 hco54        15 dih54
 h   30 hc55       29 hcc55        28 dih55
 h   30 hc56       29 hcc56        28 dih56
 h   30 hc57       29 hcc57        28 dih57
```

```
cc2          1.513568
oc3          1.444767
occ3         111.519
co4          1.327694
coc4         117.583
dih4          83.237
oc5          1.206558
oco5         124.897
dih5          -0.710
cc6          1.509592
cco6         110.390
dih6        -179.110
nc7          1.441803
ncc7         110.731
dih7        -164.527
cn8          1.337946
cnc8         121.010
dih8         150.748
oc9          1.231996
ocn9         123.945
dih9           6.932
cc10         1.499751
ccn10        115.002
dih10       -173.250
cc11         1.332471
ccc11        124.850
dih11         89.616
cc12         1.502295
ccc12        122.595
dih12         -2.292
nc13         1.345806
ncc13        117.017
dih13         45.910
cn14         1.438250
cnc14        121.819
dih14       -171.997
cc15         1.521185
ccn15        112.400
dih15         79.018
oc16         1.205027
occ16        124.635
dih16         -6.268
cc17         1.515612
```

ccc17	120.896
dih17	-172.987
cc18	1.528133
ccc18	109.277
dih18	73.202
nc19	1.353444
ncc19	114.538
dih19	64.753
cn20	1.436290
cnc20	120.946
dih20	-160.136
cc21	1.522855
ccn21	111.861
dih21	74.019
oc22	1.207983
occ22	124.277
dih22	-8.126
oc23	1.223513
ocn23	123.423
dih23	18.003
oc24	1.324413
occ24	111.112
dih24	172.196
co25	1.450072
coc25	117.368
dih25	179.839
cc26	1.513724
cco26	111.205
dih26	-83.522
oc27	1.231238
occ27	120.361
dih27	-135.452
oc28	1.327946
occ28	110.537
dih28	174.346
co29	1.448016
coc29	117.265
dih29	-179.754
cc30	1.514997
cco30	111.450
dih30	80.772
hc31	1.086538
hcc31	119.630
dih31	5.079
hn32	1.017122
hnc32	119.322
dih32	-4.647
hc33	1.095785
hcn33	111.729
dih33	-88.974
hc34	1.090507
hcn34	109.741
dih34	30.150
hc35	1.089250
hco35	103.939
dih35	-156.513
hc36	1.090369
hco36	108.595
dih36	-40.494
hc37	1.091240
hcc37	111.109
dih37	-64.379
hc38	1.092113
hcc38	110.780
dih38	56.987
hc39	1.092751
hcc39	109.231
dih39	176.159
hn40	1.017544
hnc40	115.991
dih40	172.145

```
hc41        1.090165
hcn41       109.987
dih41      -164.737
hc42        1.091911
hcn42       110.463
dih42       -46.208
hc43        1.090190
hco43       108.457
dih43        40.024
hc44        1.088968
hco44       103.952
dih44       156.424
hc45        1.092365
hcc45       109.170
dih45      -176.371
hc46        1.092048
hcc46       110.920
dih46       -57.132
hc47        1.091679
hcc47       111.070
dih47        64.277
hc48        1.091287
hcc48       110.679
dih48      -165.508
hc49        1.088544
hcc49       110.484
dih49       -45.004
hn50        1.011243
hnc50       118.826
dih50        -4.168
hc51        1.092348
hcn51       109.990
dih51       -41.455
hc52        1.090171
hcn52       109.563
dih52      -159.596
hc53        1.088902
hco53       104.015
dih53      -158.905
hc54        1.090388
hco54       108.653
dih54       -42.637
hc55        1.092462
hcc55       110.751
dih55        56.301
hc56        1.092854
hcc56       109.051
dih56       175.294
hc57        1.091172
hcc57       111.121
dih57       -65.378

#7.2
 c
 c   1 cc2
 o    2 oc3         1 occ3
 c    3 co4         2 coc4          1 dih4
 o    4 oc5         3 oco5          2 dih5
 c    4 cc6         3 cco6          2 dih6
 n    6 nc7         4 ncc7          3 dih7
 c    7 cn8         6 cnc8          4 dih8
 o    8 oc9         7 ocn9          6 dih9
 c    8 cc10        7 ccn10         6 dih10
 c   10 cc11        8 ccc11         7 dih11
 c   11 cc12       10 ccc12         8 dih12
 n   12 nc13       11 ncc13        10 dih13
 c   13 cn14       12 cnc14        11 dih14
 c   14 cc15       13 ccn15        12 dih15
 o   15 oc16       14 occ16        13 dih16
 c   11 cc17       10 ccc17         8 dih17
 c   17 cc18       11 ccc18        10 dih18
```

```
 n   18 nc19       17 ncc19        11 dih19
 c   19 cn20       18 cnc20        17 dih20
 c   20 cc21       19 ccn21        18 dih21
 o   21 oc22       20 occ22        19 dih22
 o   18 oc23       19 ocn23        20 dih23
 o   21 oc24       20 occ24        19 dih24
 c   24 co25       21 coc25        20 dih25
 c   25 cc26       24 cco26        21 dih26
 o   12 oc27       11 occ27        10 dih27
 o   15 oc28       14 occ28        13 dih28
 c   28 co29       15 coc29        14 dih29
 c   29 cc30       28 cco30        15 dih30
 h   10 hc31       11 hcc31        17 dih31
 h    7 hn32        8 hnc32        10 dih32
 h    6 hc33        7 hcn33         8 dih33
 h    6 hc34        7 hcn34         8 dih34
 h    2 hc35        3 hco35         4 dih35
 h    2 hc36        3 hco36         4 dih36
 h    1 hc37        2 hcc37         3 dih37
 h    1 hc38        2 hcc38         3 dih38
 h    1 hc39        2 hcc39         3 dih39
 h   19 hn40       18 hnc40        23 dih40
 h   20 hc41       19 hcn41        18 dih41
 h   20 hc42       19 hcn42        18 dih42
 h   25 hc43       24 hco43        21 dih43
 h   25 hc44       24 hco44        21 dih44
 h   26 hc45       25 hcc45        24 dih45
 h   26 hc46       25 hcc46        24 dih46
 h   26 hc47       25 hcc47        24 dih47
 h   17 hc48       11 hcc48        10 dih48
 h   17 hc49       11 hcc49        10 dih49
 h   13 hn50       12 hnc50        11 dih50
 h   14 hc51       13 hcn51        12 dih51
 h   14 hc52       13 hcn52        12 dih52
 h   29 hc53       28 hco53        15 dih53
 h   29 hc54       28 hco54        15 dih54
 h   30 hc55       29 hcc55        28 dih55
 h   30 hc56       29 hcc56        28 dih56
 h   30 hc57       29 hcc57        28 dih57

cc2         1.514413
oc3         1.445941
occ3        111.119
co4         1.329440
coc4        117.847
dih4        -83.645
oc5         1.204600
oco5        124.947
dih5         -1.041
cc6         1.522023
cco6        109.892
dih6       -179.967
nc7         1.435850
ncc7        113.048
dih7       -171.523
cn8         1.356033
cnc8        121.138
dih8         77.000
oc9         1.224691
ocn9        122.027
dih9          3.614
cc10        1.486636
ccn10       114.315
dih10      -176.993
cc11        1.331289
ccc11       123.930
dih11      -175.792
cc12        1.509037
ccc12       124.468
dih12        -2.478
nc13        1.346035
```

ncc13	115.489
dih13	90.861
cn14	1.435339
cnc14	120.829
dih14	167.720
cc15	1.520836
ccn15	112.463
dih15	-75.428
oc16	1.210964
occ16	124.734
dih16	-8.933
cc17	1.514083
ccc17	121.754
dih17	178.569
cc18	1.527124
ccc18	109.616
dih18	-90.507
nc19	1.350041
ncc19	115.677
dih19	-88.470
cn20	1.436031
cnc20	120.637
dih20	168.916
cc21	1.521117
ccn21	112.585
dih21	-74.111
oc22	1.211195
occ22	124.863
dih22	-7.615
oc23	1.223968
ocn23	122.957
dih23	-9.297
oc24	1.322026
occ24	110.733
dih24	172.868
co25	1.449326
coc25	117.934
dih25	177.384
cc26	1.513464
cco26	111.142
dih26	-82.489
oc27	1.225297
occ27	120.404
dih27	-95.604
oc28	1.321842
occ28	110.760
dih28	172.218
co29	1.448212
coc29	118.052
dih29	179.714
cc30	1.513860
cco30	111.091
dih30	83.438
hc31	1.086478
hcc31	118.668
dih31	-1.587
hn32	1.006905
hnc32	119.528
dih32	-6.243
hc33	1.090924
hcn33	109.420
dih33	-162.315
hc34	1.091210
hcn34	110.171
dih34	-44.261
hc35	1.090446
hco35	108.732
dih35	39.700
hc36	1.087965
hco36	104.175
dih36	156.076

```
hc37        1.091607
hcc37       111.085
dih37        63.895
hc38        1.092314
hcc38       109.248
dih38      -176.598
hc39        1.092583
hcc39       110.737
dih39       -57.336
hn40        1.017240
hnc40       119.892
dih40      -171.510
hc41        1.090470
hcn41       110.845
dih41        47.515
hc42        1.091634
hcn42       109.358
dih42       165.983
hc43        1.090018
hco43       108.598
dih43        41.034
hc44        1.088528
hco44       103.915
dih44       157.334
hc45        1.092521
hcc45       109.126
dih45      -176.327
hc46        1.092651
hcc46       110.821
dih46       -57.209
hc47        1.090980
hcc47       111.162
dih47        64.306
hc48        1.089760
hcc48       110.153
dih48        27.377
hc49        1.092291
hcc49       110.063
dih49       147.264
hn50        1.016780
hnc50       119.722
dih50         1.469
hc51        1.091336
hcn51       109.409
dih51       164.478
hc52        1.091741
hcn52       110.796
dih52        46.071
hc53        1.088107
hco53       103.990
dih53      -156.337
hc54        1.090942
hco54       108.693
dih54       -39.998
hc55        1.092191
hcc55       110.770
dih55        57.290
hc56        1.092945
hcc56       109.181
dih56       176.450
hc57        1.090985
hcc57       111.109
dih57       -64.106

#7.3
 c
 c   1 cc2
 o    2 oc3         1 occ3
 c    3 co4         2 coc4          1 dih4
 o    4 oc5         3 oco5          2 dih5
 c    4 cc6         3 cco6          2 dih6
```

```
 n    6 nc7         4 ncc7          3 dih7
 c    7 cn8         6 cnc8          4 dih8
 o    8 oc9         7 ocn9          6 dih9
 c    8 cc10        7 ccn10         6 dih10
 c   10 cc11        8 ccc11         7 dih11
 c   11 cc12       10 ccc12         8 dih12
 n   12 nc13       11 ncc13        10 dih13
 c   13 cn14       12 cnc14        11 dih14
 c   14 cc15       13 ccn15        12 dih15
 o   15 oc16       14 occ16        13 dih16
 c   11 cc17       10 ccc17         8 dih17
 c   17 cc18       11 ccc18        10 dih18
 n   18 nc19       17 ncc19        11 dih19
 c   19 cn20       18 cnc20        17 dih20
 c   20 cc21       19 ccn21        18 dih21
 o   21 oc22       20 occ22        19 dih22
 o   18 oc23       19 ocn23        20 dih23
 o   21 oc24       20 occ24        19 dih24
 c   24 co25       21 coc25        20 dih25
 c   25 cc26       24 cco26        21 dih26
 o   12 oc27       11 occ27        10 dih27
 o   15 oc28       14 occ28        13 dih28
 c   28 co29       15 coc29        14 dih29
 c   29 cc30       28 cco30        15 dih30
 h   10 hc31       11 hcc31        17 dih31
 h    7 hn32        8 hnc32        10 dih32
 h    6 hc33        7 hcn33         8 dih33
 h    6 hc34        7 hcn34         8 dih34
 h    2 hc35        3 hco35         4 dih35
 h    2 hc36        3 hco36         4 dih36
 h    1 hc37        2 hcc37         3 dih37
 h    1 hc38        2 hcc38         3 dih38
 h    1 hc39        2 hcc39         3 dih39
 h   19 hn40       18 hnc40        23 dih40
 h   20 hc41       19 hcn41        18 dih41
 h   20 hc42       19 hcn42        18 dih42
 h   25 hc43       24 hco43        21 dih43
 h   25 hc44       24 hco44        21 dih44
 h   26 hc45       25 hcc45        24 dih45
 h   26 hc46       25 hcc46        24 dih46
 h   26 hc47       25 hcc47        24 dih47
 h   17 hc48       11 hcc48        10 dih48
 h   17 hc49       11 hcc49        10 dih49
 h   13 hn50       12 hnc50        11 dih50
 h   14 hc51       13 hcn51        12 dih51
 h   14 hc52       13 hcn52        12 dih52
 h   29 hc53       28 hco53        15 dih53
 h   29 hc54       28 hco54        15 dih54
 h   30 hc55       29 hcc55        28 dih55
 h   30 hc56       29 hcc56        28 dih56
 h   30 hc57       29 hcc57        28 dih57

cc2          1.514226
oc3          1.445263
occ3         111.322
co4          1.328050
coc4         117.575
dih4         -82.439
oc5          1.206501
oco5         124.818
dih5          -1.212
cc6          1.511530
cco6         110.283
dih6         177.038
nc7          1.439316
ncc7         110.696
dih7         172.932
cn8          1.339838
cnc8         122.551
dih8        -145.641
oc9          1.228371
```

```
ocn9        124.741
dih9         -3.424
cc10        1.501652
ccn10       113.550
dih10       177.188
cc11        1.330823
ccc11       124.375
dih11       -81.116
cc12        1.502042
ccc12       122.015
dih12        -1.243
nc13        1.345052
ncc13       116.849
dih13       -50.438
cn14        1.440528
cnc14       120.969
dih14       176.906
cc15        1.521133
ccn15       112.333
dih15       -81.112
oc16        1.205671
occ16       124.212
dih16       -25.283
cc17        1.516193
ccc17       121.419
dih17       170.369
cc18        1.529715
ccc18       109.432
dih18       -74.549
nc19        1.353337
ncc19       114.452
dih19       -63.662
cn20        1.436867
cnc20       121.643
dih20       160.993
cc21        1.522514
ccn21       111.632
dih21       -79.376
oc22        1.208915
occ22       124.071
dih22        17.223
oc23        1.223074
ocn23       123.619
dih23       -17.568
oc24        1.323927
occ24       111.428
dih24      -163.198
co25        1.450074
coc25       117.409
dih25       179.845
cc26        1.514212
cco26       111.364
dih26        83.007
oc27        1.231971
occ27       120.802
dih27       131.460
oc28        1.326763
occ28       110.926
dih28       156.162
co29        1.447387
coc29       117.261
dih29      -179.137
cc30        1.514127
cco30       110.821
dih30        82.303
hc31        1.086847
hcc31       119.988
dih31        -6.195
hn32        1.017328
hnc32       118.965
dih32         0.544
```

```
hc33        1.090190
hcn33       109.498
dih33       -25.447
hc34        1.096009
hcn34       111.828
dih34        93.986
hc35        1.089812
hco35       108.673
dih35        40.949
hc36        1.089037
hco36       104.133
dih36       157.232
hc37        1.093213
hcc37       109.242
dih37      -176.352
hc38        1.092426
hcc38       110.816
dih38       -57.100
hc39        1.091694
hcc39       111.095
dih39        64.285
hn40        1.018608
hnc40       116.061
dih40      -172.436
hc41        1.091921
hcn41       110.115
dih41        40.263
hc42        1.089692
hcn42       110.211
dih42       159.016
hc43        1.089324
hco43       103.868
dih43      -156.935
hc44        1.089621
hco44       108.294
dih44       -40.761
hc45        1.091929
hcc45       110.902
dih45        57.111
hc46        1.092221
hcc46       109.082
dih46       176.318
hc47        1.091717
hcc47       111.091
dih47       -64.365
hc48        1.088867
hcc48       110.401
dih48        43.594
hc49        1.091902
hcc49       110.839
dih49       164.073
hn50        1.009575
hnc50       119.346
dih50         4.844
hc51        1.091870
hcn51       109.065
dih51       159.332
hc52        1.089228
hcn52       109.983
dih52        41.025
hc53        1.088979
hco53       104.138
dih53      -157.438
hc54        1.089944
hco54       108.813
dih54       -40.933
hc55        1.093102
hcc55       109.219
dih55       176.906
hc56        1.092368
hcc56       110.996
```

```
dih56       -63.508
hc57        1.091700
hcc57       110.774
dih57        57.806

#7.4
 c
 c   1 cc2
 o    2 oc3         1 occ3
 c    3 co4         2 coc4          1 dih4
 o    4 oc5         3 oco5          2 dih5
 c    4 cc6         3 cco6          2 dih6
 n    6 nc7         4 ncc7          3 dih7
 c    7 cn8         6 cnc8          4 dih8
 o    8 oc9         7 ocn9          6 dih9
 c    8 cc10        7 ccn10         6 dih10
 c   10 cc11        8 ccc11         7 dih11
 c   11 cc12       10 ccc12         8 dih12
 n   12 nc13       11 ncc13        10 dih13
 c   13 cn14       12 cnc14        11 dih14
 c   14 cc15       13 ccn15        12 dih15
 o   15 oc16       14 occ16        13 dih16
 c   11 cc17       10 ccc17         8 dih17
 c   17 cc18       11 ccc18        10 dih18
 n   18 nc19       17 ncc19        11 dih19
 c   19 cn20       18 cnc20        17 dih20
 c   20 cc21       19 ccn21        18 dih21
 o   21 oc22       20 occ22        19 dih22
 o   18 oc23       19 ocn23        20 dih23
 o   21 oc24       20 occ24        19 dih24
 c   24 co25       21 coc25        20 dih25
 c   25 cc26       24 cco26        21 dih26
 o   12 oc27       11 occ27        10 dih27
 o   15 oc28       14 occ28        13 dih28
 c   28 co29       15 coc29        14 dih29
 c   29 cc30       28 cco30        15 dih30
 h   10 hc31       11 hcc31        17 dih31
 h    7 hn32        8 hnc32        10 dih32
 h    6 hc33        7 hcn33         8 dih33
 h    6 hc34        7 hcn34         8 dih34
 h    2 hc35        3 hco35         4 dih35
 h    2 hc36        3 hco36         4 dih36
 h    1 hc37        2 hcc37         3 dih37
 h    1 hc38        2 hcc38         3 dih38
 h    1 hc39        2 hcc39         3 dih39
 h   19 hn40       18 hnc40        23 dih40
 h   20 hc41       19 hcn41        18 dih41
 h   20 hc42       19 hcn42        18 dih42
 h   25 hc43       24 hco43        21 dih43
 h   25 hc44       24 hco44        21 dih44
 h   26 hc45       25 hcc45        24 dih45
 h   26 hc46       25 hcc46        24 dih46
 h   26 hc47       25 hcc47        24 dih47
 h   17 hc48       11 hcc48        10 dih48
 h   17 hc49       11 hcc49        10 dih49
 h   13 hn50       12 hnc50        11 dih50
 h   14 hc51       13 hcn51        12 dih51
 h   14 hc52       13 hcn52        12 dih52
 h   29 hc53       28 hco53        15 dih53
 h   29 hc54       28 hco54        15 dih54
 h   30 hc55       29 hcc55        28 dih55
 h   30 hc56       29 hcc56        28 dih56
 h   30 hc57       29 hcc57        28 dih57

cc2         1.513884
oc3         1.447250
occ3        111.329
co4         1.330139
coc4        117.152
dih4         81.675
oc5         1.204708
```

oco5	124.752
dih5	0.702
cc6	1.520790
cco6	110.726
dih6	179.825
nc7	1.437897
ncc7	112.062
dih7	175.435
cn8	1.348888
cnc8	120.695
dih8	-79.395
oc9	1.228302
ocn9	123.233
dih9	-11.833
cc10	1.495879
ccn10	117.953
dih10	170.544
cc11	1.334040
ccc11	129.069
dih11	-47.587
cc12	1.510098
ccc12	123.162
dih12	7.296
nc13	1.339630
ncc13	114.630
dih13	-121.509
cn14	1.440849
cnc14	122.431
dih14	175.595
cc15	1.521654
ccn15	114.372
dih15	-98.396
oc16	1.208925
occ16	122.634
dih16	157.214
cc17	1.515742
ccc17	119.779
dih17	-172.218
cc18	1.523109
ccc18	113.010
dih18	89.238
nc19	1.344793
ncc19	115.626
dih19	-146.556
cn20	1.440778
cnc20	120.949
dih20	-173.778
cc21	1.521527
ccn21	115.454
dih21	69.543
oc22	1.207257
occ22	121.950
dih22	-164.672
oc23	1.228596
ocn23	121.655
dih23	4.952
oc24	1.324817
occ24	113.051
dih24	16.551
co25	1.446464
coc25	117.797
dih25	178.777
cc26	1.514169
cco26	111.103
dih26	-85.060
oc27	1.232218
occ27	121.358
dih27	60.364
oc28	1.320548
occ28	112.430
dih28	-25.273

co29	1.443599
coc29	117.520
dih29	177.682
cc30	1.509051
cco30	107.300
dih30	-171.534
hc31	1.086570
hcc31	118.251
dih31	-0.923
hn32	1.014875
hnc32	117.137
dih32	13.028
hc33	1.090072
hcn33	110.248
dih33	41.868
hc34	1.091495
hcn34	109.581
dih34	160.276
hc35	1.089100
hco35	104.145
dih35	-157.999
hc36	1.091033
hco36	108.646
dih36	-41.741
hc37	1.091350
hcc37	110.877
dih37	58.245
hc38	1.092539
hcc38	109.249
dih38	177.592
hc39	1.091865
hcc39	111.029
dih39	-63.064
hn40	1.006941
hnc40	119.492
dih40	175.913
hc41	1.091703
hcn41	108.727
dih41	-169.814
hc42	1.090144
hcn42	110.471
dih42	-52.000
hc43	1.090861
hco43	108.678
dih43	38.384
hc44	1.088998
hco44	104.080
dih44	154.667
hc45	1.090917
hcc45	111.094
dih45	64.119
hc46	1.092437
hcc46	109.171
dih46	-176.519
hc47	1.092262
hcc47	110.758
dih47	-57.341
hc48	1.094783
hcc48	110.473
dih48	-151.363
hc49	1.090850
hcc49	109.352
dih49	-33.809
hn50	1.019860
hnc50	117.030
dih50	4.214
hc51	1.093482
hcn51	109.622
dih51	141.240
hc52	1.088564
hcn52	109.554

```
dih52        22.881
hc53        1.090773
hco53       108.041
dih53       -51.001
hc54        1.092737
hco54       108.432
dih54        67.089
hc55        1.091535
hcc55       111.200
dih55       -60.421
hc56        1.092553
hcc56       110.383
dih56        60.617
hc57        1.092236
hcc57       109.356
dih57       179.940
```

Table S1 – Calculated Gibbs free energy values (column 3) of the representative conformations of **6** and **7** compounds obtained by DFT minimum energy optimization of the CREST structures. All calculated geometries correspond to local minima by vibrational frequencies analyses showing no negative eigenvalue of the corresponding hessian matrices, i. e. no imaginary frequency. The Gibbs free energy variations respect to the global minimum, i.e. **6.1**, and the corresponding Boltzmann population (percentage) of each conformer are also reported (column 5 and 6, respectively).

Compound	Conformation	G^0 [hartree]	#imaginary frequencies	ΔG^0 [kcal/mol]	%population
**6**	1	-1543.13927	0	0.0	44.89
	2	-1543.138663	0	0.4	23.61
	3	-1543.137667	0	1.0	8.23
**7**	1	-1543.132108	0	4.5	0.02
	2	-1543.138646	0	0.4	23.19
	3	-1543.132661	0	4.1	0.04
	4	-1543.131694	0	4.8	0.01

Full gaussian 16 reference

Gaussian 16, Revision B.01, Frisch, M. J.; Trucks, G. W.; Schlegel, H. B.; Scuseria, G. E.; Robb, M. A.; Cheeseman, J. R.; Scalmani, G.; Barone, V.; Petersson, G. A.; Nakatsuji, H.; Li, X.; Caricato, M.; Marenich, A. V.; Bloino, J.; Janesko, B. G.; Gomperts, R.; Mennucci, B.; Hratchian, H. P.; Ortiz, J. V.; Izmaylov, A. F.; Sonnenberg, J. L.; Williams-Young, D.; Ding, F.; Lipparini, F.; Egidi, F.; Goings, J.; Peng, B.; Petrone, A.; Henderson, T.; Ranasinghe, D.; Zakrzewski, V. G.; Gao, J.; Rega, N.; Zheng, G.; Liang, W.; Hada, M.; Ehara, M.; Toyota, K.; Fukuda, R.; Hasegawa, J.; Ishida, M.; Nakajima, T.; Honda, Y.; Kitao, O.; Nakai, H.; Vreven, T.; Throssell, K.; Montgomery, J. A., Jr.; Peralta, J. E.; Ogliaro, F.; Bearpark, M. J.; Heyd, J. J.; Brothers, E. N.; Kudin, K. N.; Staroverov, V. N.; Keith, T. A.; Kobayashi, R.; Normand, J.; Raghavachari, K.; Rendell, A. P.; Burant, J. C.; Iyengar, S. S.; Tomasi, J.; Cossi, M.; Millam, J. M.; Klene, M.; Adamo, C.; Cammi, R.; Ochterski, J. W.; Martin, R. L.; Morokuma, K.; Farkas, O.; Foresman, J. B.; Fox, D. J. Gaussian, Inc., Wallingford CT, 2016.
